# Epigenetic modification of CD4^+^ T cells into Tregs by 5-azacytidine as cellular therapeutic for atherosclerosis treatment

**DOI:** 10.1038/s41419-024-07086-7

**Published:** 2024-09-20

**Authors:** Ling Zhu, Zhongwei Liu, Qianwei Cui, Gongchang Guan, Rutai Hui, Xiqiang Wang, Junkui Wang, Yong Zhang, Xu Zhu

**Affiliations:** 1 Department of Cardiology, Shaanxi Provincial People’s Hospital, Xi’an, Shaanxi China; 2 Department of Cardiology, The Third Affiliated Hospital of Xi’an Jiaotong University, Xi’an, Shaanxi China; 3 Shaanxi Provincial Traditional Chinese Medicine Key Laboratory, Xi’an, Shaanxi China; 4 Department of Cardiology, State Key Laboratory of Cardiovascular Disease, Fuwai Hospital, National Center for Cardiovascular Diseases, Chinese Academy of Medical Sciences and Peking Union Medical College, Beijing, China; 5 Department of Cardiology, The First Affiliated Hospital of Nanjing Medical University, Jiangsu Province Hospital, Nanjing, China

**Keywords:** Immunology, Cell biology

## Abstract

Recent research has explored the potential of the demethylating drug 5-azacytidine (Aza) as therapy for a range of diseases. However, the therapeutic efficacy of Aza for patients of atherosclerosis remains unclear. This study investigates the therapeutic application of Aza to atherosclerosis in order to elucidate the underlying mechanisms. We generated induced Tregs (iTregs) from CD4^+^ T cells by using Aza in vitro, and this was followed by the intravenous infusion of iTregs for the treatment of atherosclerosis. The adoptive transfer of Aza-iTreg significantly increased peripheral blood Treg cells, suppressed inflammation, and attenuated atherosclerosis in ApoE^−/−^ mice. Furthermore, we observed a notable demethylation of the Forkhead box P3 (Foxp3)-regulatory T cell-specific demethylated region (TSDR) and an upregulation of Foxp3 expression in the CD4^+^ T cells in the spleen of the ApoE^−/−^ mice following the transfer of Aza- iTregs. We also demonstrated that Aza converted naive CD4^+^ T cells into Tregs by DNA methyltransferase 1 (Dnmt1)-mediated Foxp3-TSDR demethylation and the upregulation of Foxp3 expression. Conversely, the overexpression of Dnmt1 in the CD4^+^ T cells attenuated the Aza-induced Foxp3-TSDR demethylation and upregulation of Foxp3 expression. Our results reveal that Aza converts naive CD4^+^ T cells into functional Tregs by inhibiting Dnmt1, and the transfer of Aza-iTregs suppresses atherosclerosis in mice.

## Introduction

Atherosclerosis is a persistent inflammatory condition that primarily affects the arterial wall and is the primary instigator of cardiovascular disorders worldwide [1, 2]. T-cell-mediated pathogenic immunological responses are known to play a crucial role in atherogenesis [2, 3], where this involves Th1, Th2, and natural killer T cells. Extensive experimental investigations have consistently demonstrated that Th1 cells form the dominant subset of CD4^+^ T cells present in atherosclerotic lesions across murine and human models [4]. Multiple independent groups have also provided compelling evidence that Th1-driven responses have detrimental effects on the atherosclerotic process [5, 6]. Regulatory T cells (Tregs) constitute an identifiable subset of T cells that play a critical role in maintaining immunological equilibrium and promoting immunologic tolerance through their capacity to suppress the activity of effector T cells [7]. The transcription factor Forkhead box P3 (Foxp3) serves as a pivotal regulator in the formation and function of CD4^+^CD25^+^ Tregs [8]. Tregs are involved in defending against the initiation and progression of atherosclerosis [9, 10], and thus offer promise as a potential treatment for the condition. Several studies have demonstrated that the exogenous infusion of Tregs inhibits and reverses atherosclerosis, and prevents it as well [10–12]. However, Tregs are scarce, accounting for only 5% to 10% of CD4^+^ T cells [7]. Their allogeneic infusion may have limited clinical application due to rejection. Cell therapy for atherosclerosis is a feasible, safe, and effective approach, but obtaining a sufficient number of Tregs and avoiding immunological rejection remain significant challenges in this regard.

5-azacytidine (Aza) is a well-known inhibitor of DNA methyltransferases (Dnmts). Once it has been taken up by cells, Aza undergoes phosphorylation to form 5-aza-2′-deoxycytidine-5′-triphosphate, which is subsequently integrated into the DNA to inhibit the activity of Dnmts [13]. Recent research has explored the potential of demethylating drugs as therapy for a range of diseases [14–17]. Notably, past research has demonstrated that Aza can induce the transformation of effector T cells into Tregs by inhibiting the methylation of the Foxp3 gene [18–20]. Our previous investigations have revealed a significant association between the progression of atherosclerosis and the increased methylation of the Foxp3-TSDR (regulatory T-cell-specific demethylated region) [21, 22]. However, the therapeutic effect of Aza on atherosclerosis has yet to be determined. The primary objective of this study is to induce the generation of Tregs from CD4^+^ T cells by using Aza in vitro, followed by the intravenous infusion of induced Tregs (iTregs) to treat atherosclerosis, in order to elucidate the underlying mechanisms involved.

## Methods

### Animals

ApoE^−/−^ mice (C57BL/6J background) were transitioned from a standard chow diet to a western diet (WD) at six weeks of age. Preceding this dietary shift, the mice had been weaned at four weeks and sustained on the standard chow diet. The WD used in this investigation comprised 21% fat and 0.15% cholesterol. A cohort of 48 male mice, aged six weeks, was randomly divided into eight groups. Four of these groups were given the WD for an eight-week period, while the remaining four groups were subjected to a 12-week WD feeding. The mice in the eight-week groups were administered the WD for eight weeks (from six to 14 weeks of age), and received tail vein injections of saline, either with or without cells, at ten weeks of age (200 μl; 1 × 10^6^ cells per injection). In parallel, the mice in the 12-week groups consumed the WD for 12 weeks (from six to 18 weeks of age), and received tail vein injections of saline, with or without cells, at ten and 14 weeks of age (200 μl; 1 × 10^6^ cells per injection). The experimental setup for the animal studies is delineated in Fig. S1 in the supplementary files. To collect the peripheral blood, the animals were anesthetised via isoflurane inhalation (3% for induction and 1.5% for maintenance at 0.4 L/min) and positioned in a supine orientation. Following euthanasia by cervical dislocation, perfusion was performed on them, and the spleen and aorta were subsequently extracted.

### Cell isolation and adoptive transfer study

Naive CD4^+^ T cells (Tn) and CD4^+^ CD25^+^ T cells (Treg) were extracted from the spleens of C57BL/6J mice by using the CD4^+^ T Cell Isolation Kit and CD4^+^ CD25^+^ Regulatory T Cell Isolation Kit (Miltenyi Biotech, Bergisch Gladbach, Germany), and by following the instructions provided by the manufacturer. The isolated Tn and Tregs were cultured in a six-well plate at a density of 2 × 10^6^ cells per well by using the primary T lymphocyte culture system (Wanwu Technology, Hefei, China). In the Aza-induced Treg group (Aza-iTreg), the Tn were stimulated with a DNA methyltransferase inhibitor (Aza) at a concentration of 5 μM. The isolated cells were incubated for 48 h at 37 °C in a controlled atmosphere with 5% CO_2_. Following this, the cells were injected into the mice through the tail vein (four groups: Tn, saline, Aza-iTreg, Treg; 200 μL, 1 × 10^6^ cells per injection) (Fig. S1).

### Evaluation of atherosclerotic lesions

The aortic root lesions were evaluated by following the established protocols described in our previous study [22]. A single slide was used to harvest every tenth section (three sections per slide). The average areas of the lesions were subsequently calculated based on three sections for each mouse. The entire aorta was isolated, and the lipid deposits were quantified by using oil red O staining. The lipids were also visualized by using oil red O staining (Sigma), and the collagen was labelled by using the Sirius Red staining method (Sigma). The digital representations of the lipid and collagen sections were acquired by using the Olympus BX53 imaging system and the Nikon Eclipse Ci imaging system, respectively. The macrophage abundance was quantified through immunofluorescence staining by using a CD68 monoclonal antibody (CD68, 1:100, Affinity Biosciences). The sections were then treated with Alexa 555-conjugated secondary antibodies (Abcam). The captured images were observed by using an Olympus microscope, model BX53. The distribution of Foxp3 in the aortic root lesions was observed by double-label immunofluorescence staining (CD4, 1:100, Abcam) (Foxp3, 1:100, Santa). The sections were subsequently subjected to additional incubation, with the secondary antibodies labelled with Alexa 555 (Abcam) to detect CD4 and with Alexa 488 (Abcam) to detect Foxp3. The images were captured by using a Nikon DS-Fi3 microscope and labelled with DAPI to highlight the nuclei.

### Flow cytometric analysis of CD4^+^ Foxp3^+^

To evaluate the proportion of CD4^+^ Foxp3^+^ cells within peripheral blood mononuclear cells (PBMCs) obtained from the ApoE^−/−^ mice, we used BD Pharmingen antibodies: specifically, CD4 (CD4 FITC, Catalogue No: 11-0041-82, eBioscience) and Foxp3 (Foxp3 PE, Catalogue No: 12-5773-82, eBioscience). A flow cytometer (Beckman Coulter) was used in accordance with the manufacturer’s guidelines, and the analysis was performed by using CytoFLEX and CytoExpert software (Beckman Coulter).

### Bisulphite sequencing

To assess the status of methylation of the Foxp3 gene TSDR in both the CD4^+^ T cells and the CD4^+^ CD25^+^ T cells, we conducted bisulphite sequencing according to the methods outlined in our previous investigation [23]. The primer sequences used to analyse the methylation of Foxp3-TSDR, along with the corresponding sequences of the mouse Foxp3-TSDR, are detailed in Table S1 (Supplementary Materials, Table S1).

### Quantitative RT-PCR

The total RNA was isolated from the tissue in the spleen of the mice—specifically, the Tn and Treg cells were isolated—in accordance with the manufacturer’s instructions. Extraction was performed by using Trizol (Aidlab Biotechnologies, Beijing, China). The obtained RNA was then reverse-transcribed into cDNA by using the PrimeScript RT reagent Kit (Takara Biotechnology, Dalian, China). SYBR green-based RT-PCR was subsequently conducted (Vazyme, Nanjing, China), with either GAPDH or β-actin serving as internal control for the normalisation of gene expression. The samples were subjected to initial denaturation at 95 °C for 10 min, followed by 40 cycles of amplification at 95 °C for 30 s and annealing/extension at 60 °C for 30 s. The relative expression levels were determined by using the 2^—△△Ct^ method. Detailed information on the specific amplification sequence used in the quantitative RT-PCR is provided in Table S2 of the Supplementary Materials.

### Western blotting

The mouse Tn and Treg, once isolated from the spleen tissue, were subjected to lysis by employing the RIPA lysis buffer system (Beyotime Biotechnology, Shanghai, China) post homogenization. The total protein concentration was determined by using a BCA kit from Beyotime Biotechnology. Subsequently, 40 μg of the protein samples were subjected to SDS-PAGE analysis. Following protein separation, the PVDF membranes were blocked with a blocking solution (provided by Beyotime Biotechnology) for 2 h at room temperature. Specific antibodies—Dnmt 1 (1:2000, Cell Signaling), Dnmt 3a (1:2000, Abcam), Dnmt3b (1:2000, Abcam), Foxp3 (1:1000, Affinity), Gapdh (1:1000, Abcam), and β-actin (1:5000, Affinity)—were subsequently incubated with the membranes. They were incubated at 4 °C for 10 h and were then washed five times with TBST. HRP-conjugated secondary antibodies (diluted 1:10,000, Boster, Wuhan, China) were subsequently added and incubated at 37 °C for 2 h and were then washed five times with TBST. Finally, the membranes were visualized by using an ECL kit (Thermo) and detected by using the Multiskan SkyHigh System (Thermo). BandScan software was used to measure the band intensities.

### Enzyme-linked immunosorbent assay

We used commercially available ELISA kits (Elabscience, Wuhan, China) to assess the concentrations of TGF-β, IL-10, IL-1β, and IFN-γ in the culture media of the isolated Tn and Treg cells. These selected kits exhibited inter-assay and intra-assay coefficients of variation below 10% for all measured analytes, including TGF-β, IL-10, IL-1β, and IFN-γ. To analyse the ApoE^−/−^ mouse plasma, we isolated it through centrifugation and stored it at −80 °C. Subsequently, the concentrations of TGF-β, IL-10, IL-1β, and IFN-γ in the ApoE^−/−^ mouse plasma were determined by using ELISA kits sourced from Elabscience, Wuhan, China, and by following the manufacturer’s instructions. The observed inter-assay and intra-assay coefficients of variation were consistently below 10% for all measured analytes. Moreover, in the Dnmt1 overexpression assay, we applied commercially available ELISA kits (Fine Biotech, Wuhan, China) to quantify the levels of TGF-β, IL-10, IL-1β, and IFN-γ in the culture medium. The inter-assay coefficients of variation of TGF-β, IL-10, IL-1β, and IFN-γ were confirmed to be below 10%, while those of all measured cytokines were consistently below 8%.

### Chromatin immunoprecipitation (ChIP) assay

We conducted ChIP to evaluate the enrichment of Dnmt1, Dnmt3a, and Dnmt3b in the TSDR region of the Foxp3 locus in the CD4^+^ T cells that were and were not induced with Aza. The ChIP assay was performed by using the EpiQuik™ Chromatin Immunoprecipitation Kit from Epigentek. The primer sequences and mouse Foxp3-TSDR sequences used for ChIP are listed in Table S3 (Supplementary Materials).

### Construction, packaging, and purification of Ad-ZsGreen-mDnmt1

#### Construction of Ad-ZsGreen-mDnmt1

The murine Dnmt1 mRNA sequence was obtained from GenBank. The vector pDC316-mCMV-ZsGreen was digested and linearised by using NotI and HindIII enzymes. The mDnmt1 sequence is provided in Table S4, while the vector structure is illustrated in Fig. S2A (Supplementary Materials). The resultant products, in conjunction with JM109 competent cells, were subsequently cultured on an LB medium with ampicillin. Post-cultivation, the recombinant plasmids were extracted by using a plasmid preparation kit from TransGen Biotech, and were subjected to meticulous verification through DNA sequencing.

#### Ad-ZsGreen-mDnmt1 packaging and purification

HEK 293 cells, cultivated under a humidified atmosphere at 37 °C, were nurtured in DMEM supplemented with 10% FBS, 5% CO_2_, and 95% air. Once a confluence exceeding 80% had been attained, the transfection of the pDC316-mCMV-ZsGreen-mDnmt1 plasmids was initiated. Successful transduction was confirmed by observing fluorescence, as shown in Fig. S2B (Supplementary Materials). Following a 48-h packaging period, the viral supernatant was extracted through centrifugation, and the vectors were subsequently purified by using the ViraBind™ Adenovirus Purification Kit from Cell Biolabs. Viral titer determination was carried out by using quantitative PCR (qPCR). The CD4^+^ T cells were infected with the Ad-ZsGreen-mDnmt1 adenovirus encoding the complementary DNA (cDNA) of Dnmt1 (AdDnmt1).

### Statistical analysis

The data were presented by following the format of mean ± SEM (standard error of the mean). Statistical comparisons between the groups were conducted by using *t*-tests. In instances where three groups or more were involved, a one-way analysis of variance (ANOVA) was employed, and the LSD test was subsequently applied to differentiate the means of treatment. A significance threshold of *P* < 0.05 was adopted to establish statistical significance. All statistical analyses were conducted by using PASW Statistics 20.0 software.

## Results

### Adoptive transfer of Aza-iTreg attenuated atherosclerosis in ApoE^−/−^ mice

**Fig. 1:**
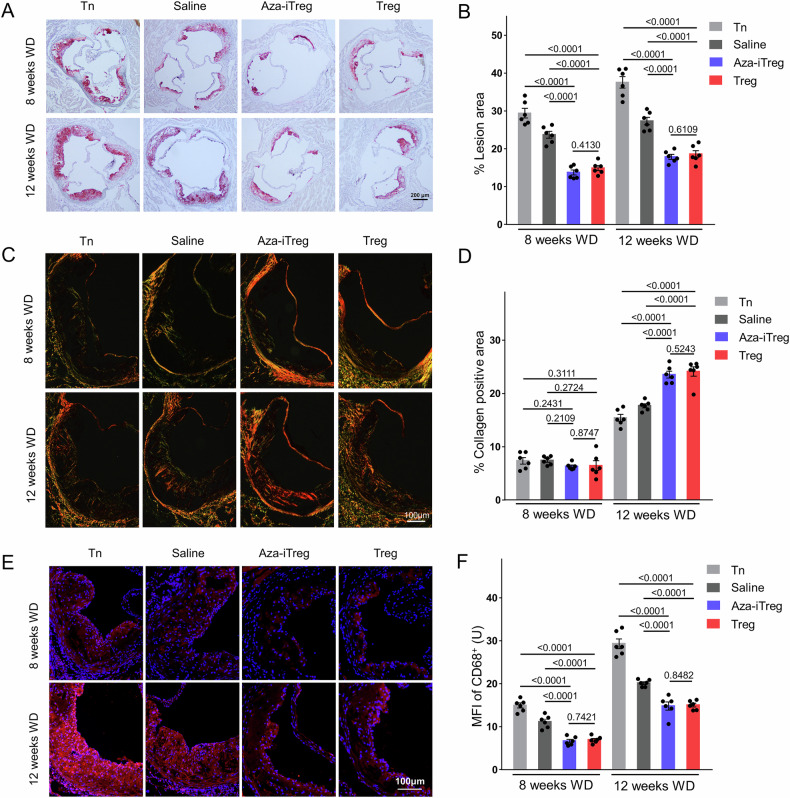
Adoptive transfer of Aza-iTreg reduced atherosclerosis in ApoE^−/−^ mice. **A** The area fraction of the lesion was measured by using oil red O staining (magnification ×40). **B** Quantitative analysis was conducted to determine the area of the aortic root AS lesion, with three sections per mice (*n* = 6). **C** Sirius Red staining was applied to evaluate the collagen content (magnification ×100). **D** Quantitative analysis was performed to measure the collagen content of the lesions, with three sections per mice (*n* = 6). **E** The immunofluorescence staining method was used to evaluate the content of macrophages (CD68; magnification ×200). **F** The macrophage content in the lesion was quantified by measuring the mean fluorescence intensity of CD68 within the lesion, with three sections per mice (*n* = 6). The data are presented as the mean ± SEM. The values on the horizontal lines in the figure are the *P* values between the two groups. Abbreviations: Aza 5-azacytidine, MFI mean fluorescence intensity, Tn naive CD4^+^ T cell, Treg regulatory T cell, WD western diet.

Isolated Tn cells were cultured with Aza (5 µM) for 48 h (Aza-iTreg) and were then injected into the mice through the tail vein. Prior to injection, cell viability was assayed by using trypan blue staining (Fig. S3). Aza-iTreg had a significant attenuating effect on the area of aortic root lesions in the ApoE^−/−^ mice subjected to a WD for eight and 12 weeks in comparison with the Tn cell transfer and the control groups (Fig. 1A, B). The transfer of normal Tregs from the C57BL/6 J mice also significantly reduced the size of the atherosclerotic lesions after eight weeks of the WD, and they exhibited a corresponding reduction after 12 weeks (Fig. 1A, B). No significant difference was observed between Aza-iTreg transfer and Treg transfer in terms of the reduction in lesion size (Fig. 1A, B). Furthermore, we evaluated the impact of Aza-iTreg transfer on the overall aortic lesions in the mice, and the results demonstrated a significant reduction in the burden of lesions throughout the aorta following Aza-iTreg transfer (Fig. S4). Although the collagen content in the lesions remained unaffected by Aza-iTreg transfer and Treg transfer after eight weeks of the WD, both treatments significantly increased collagen content in the lesions after 12 weeks (Fig. 1C, D). Furthermore, both Aza-iTreg transfer and Treg transfer resulted in a significant reduction in macrophage content within the aortic root lesions after both eight and 12 weeks of the WD, compared with the Tn cell transfer and the control groups (Fig. 1E, F). There was no significant difference between Aza-iTreg transfer and Treg transfer in terms of attenuating macrophage infiltration (Fig. 1E, F). These results suggest that Aza-iTreg transfer ameliorated the development of accelerated AS and contributed to lesion stabilization.

### Adoptive transfer of Aza-iTreg increased the proportion of Tregs and suppressed inflammatory in ApoE^−/−^ mice

**Fig. 2:**
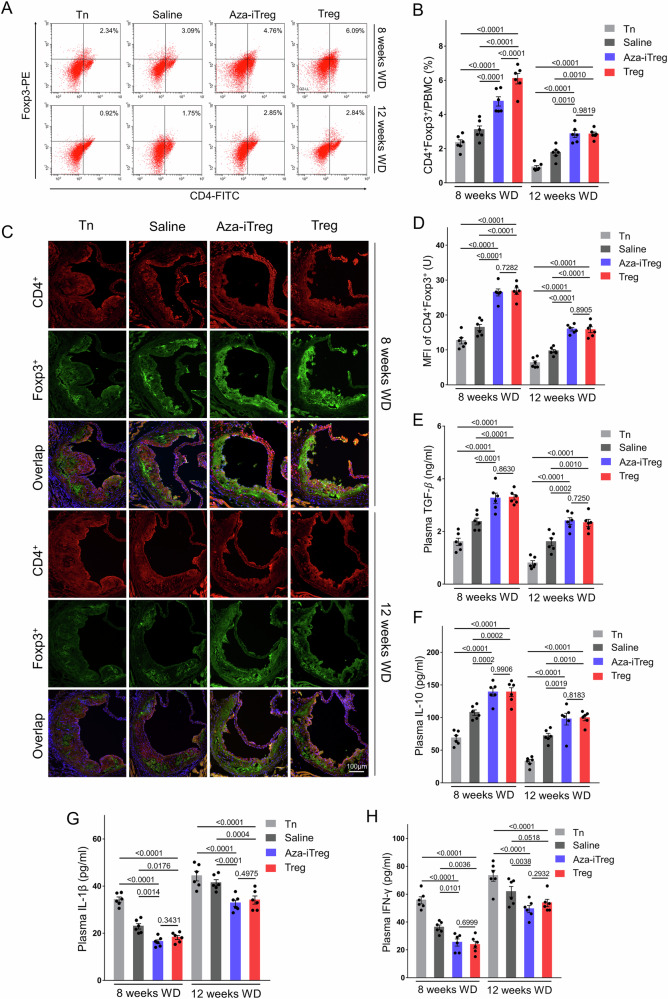
Adoptive transfer of Aza-iTreg enhanced Treg percentages and suppressed inflammatory cytokine levels in ApoE^−/−^ mice. **A** Representative FACS results depicting Tregs (CD4^+^Foxp3^+^ T cells) from a single mouse across the eight experimental groups. The numbers indicate the percentage of cells in the respective quadrants (CD4^+^Foxp3^+^/PBMC). **B** The results of statistical analysis, showing proportions of peripheral blood Tregs in the eight groups (*n* = 6). **C** Immunofluorescence staining was applied to assess the content of CD4+Foxp3+ cells in the aortic root lesions (magnification ×100). **D** Quantification of CD4^+^Foxp3^+^ cell content in the aortic root lesions by measuring the mean fluorescence intensity within the lesions (three sections per mice, *n* = 6). **E** Plasma concentrations of TGF-β in the eight groups (*n* = 6). **F** Plasma concentrations of IL-10 in the eight groups (*n* = 6). **G** Plasma concentrations of IL-1β in the eight groups (*n* = 6). **H** Plasma concentrations of IFN-γ in the eight groups (*n* = 6). The data are presented as the mean ± SEM. The values on the horizontal lines in the figure are the *P* values between the two groups. Abbreviations: Aza 5-azacytidine, MFI mean fluorescence intensity, PBMC peripheral blood mononuclear cell, Tn naive CD4^+^ T cell, Treg regulatory T cell, WD western diet.

After eight weeks of the WD, both Aza-iTreg transfer and Treg transfer showed a significant increase in the percentages of CD4^+^ Foxp3^+^ cells in the peripheral blood of ApoE^−/−^ mice, compared with the Tn cell transfer and the control groups. This increase was particularly prominent in the Treg transfer group. Similarly, after 12 weeks of the WD, both Aza-iTreg transfer and Treg transfer resulted in a significant increase in CD4^+^ Foxp3^+^ cells in the peripheral blood. There was no difference between Aza-iTreg transfer and Treg transfer in terms of the proportion of peripheral blood Tregs (Fig. 2A, B). The content of CD4^+^ Foxp3^+^ cells in the aortic root lesions was also evaluated by using immunofluorescence. After eight and 12 weeks of the WD, both Aza-iTreg transfer and Treg transfer significantly increased the content of CD4^+^ Foxp3^+^ cells in the aortic root lesions compared with the Tn cell transfer and the control groups (Fig. 2C, D). No significant difference was observed between Aza-iTreg transfer and Treg transfer in terms of increasing CD4^+^ Foxp3^+^ cells in the aortic root lesions.

Furthermore, compared with the Tn cell transfer and the control groups, both Aza-iTreg transfer and Treg transfer led to a significant upregulation of the plasma concentrations of TGF-β (Fig. 2) and IL-10 (Fig. 2F) in the mice that had been on the WD for eight and 12 weeks. There was no difference between Aza-iTreg transfer and Treg transfer in terms of plasma TGF-β and IL-10 concentrations (Fig. 2E, F). On the contrary, they led to a significant reduction in the plasma concentrations of IL-1β (Fig. 2G) and IFN-γ (Fig. 2H) in the mice that had been on the WD for eight and 12 weeks, in comparison with the Tn cell transfer and the control groups. No significant difference was observed between Aza-iTreg transfer and Treg transfer in terms of suppressing IL-1β and IFN-γ (Fig. 2G, H).

### Adoptive transfer of Aza-iTreg reduced Foxp3-TSDR methylation levels, and enhanced Foxp3 expression in CD4^+^ T cells from the spleen of ApoE^−/−^ mice

**Fig. 3:**
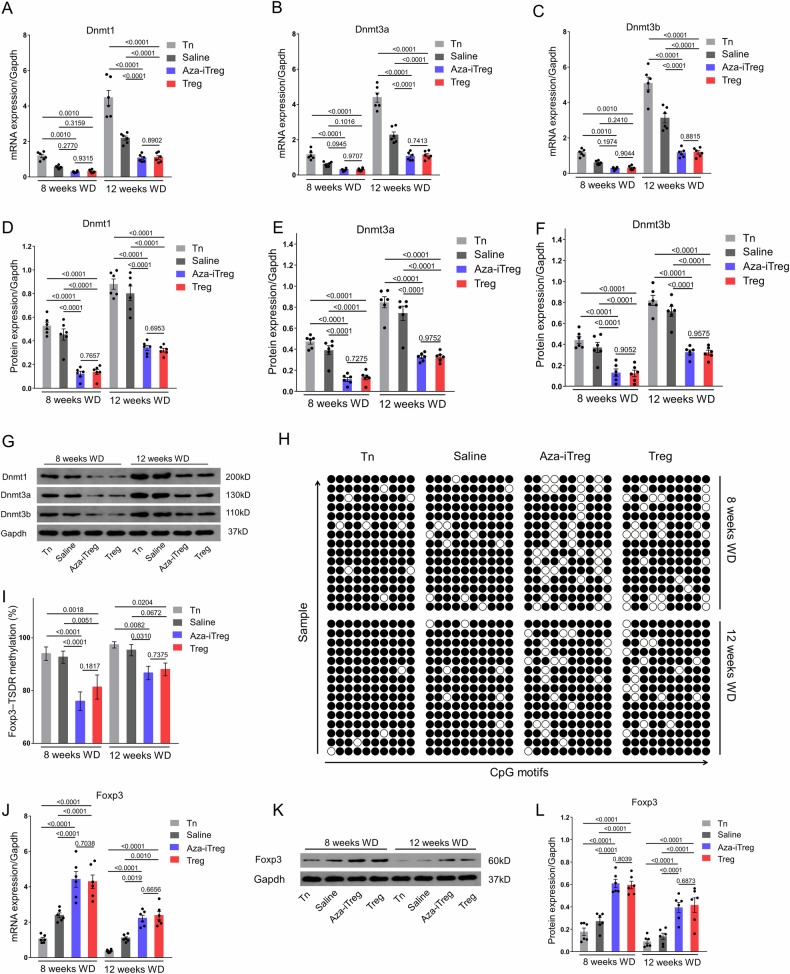
Adoptive transfer of Aza-iTreg reduced methylation levels of Foxp3-TSDR and the expression of Dnmts, while enhancing the Foxp3 expression of CD4^+^ T cells in the spleen of ApoE^−/−^ mice. **A** The levels of mRNA expression of Dnmt1 in CD4^+^ T cells from the spleen of ApoE^−/−^ mice (*n* = 6, repeated three times). **B** The levels of mRNA expression of Dnmt3a in CD4^+^ T cells from the spleen of ApoE^−/−^ mice (*n* = 6, repeated three times). **C** The levels of mRNA expression of Dnmt3b in CD4^+^ T cells from the spleen of ApoE^−/−^ mice (*n* = 6, repeated three times). **D** The protein level of Dnmt1 in CD4^+^ T cells from the spleen of ApoE^−/−^ mice (*n* = 6). **E** The protein level of Dnmt3a in CD4^+^ T cells from the spleen of ApoE^−/−^ mice (*n* = 6). **F** The protein level of Dnmt3b in CD4^+^ T cells from the spleen of ApoE^−/−^ mice (*n* = 6). **G** Western blot analysis, showing the levels of expression of Dnmt1, Dnmt3a, and Dnmt3b in CD4^+^ T cells from the spleen of ApoE^−/−^ mice. **H** Methylation status of ten individual CpG motifs within the Foxp3-TSDR, represented by white (demethylation) and black (methylation) circles. Five repetitions of sequencing per mouse. **I** Results of statistical analysis, demonstrating the methylation status of the Foxp3-TSDR in CD4^+^ T cells from the spleen of ApoE^−/−^ mice (*n* = 3, replicated five times). **J** The levels of mRNA expression of Foxp3 in CD4^+^ T cells from the spleen of ApoE^−/−^ mice (*n* = 6, repeated three times). **K** Western blot analysis, showing the level of expression of Foxp3 in CD4^+^ T cells from the spleen of ApoE^−/−^ mice. **L** The protein level of Foxp3 in CD4^+^ T cells from the spleen of ApoE^−/−^ mice (*n* = 6). The data are presented as the mean ± SEM. The values on the horizontal lines in the figure are the *P* values between the two groups. Abbreviations: Aza 5-azacytidine, Dnmt DNA methyltransferase, Foxp3 Forkhead box P3, Tn naive CD4^+^ T cell, Treg regulatory T cell, TSDR Treg-specific demethylated region, WD western diet.

CD4^+^ T cells were derived from the spleen of ApoE^−/−^ mice and isolated using the CD4^+^ T Cell Isolation Kit through magnetic cell separation. Subsequent to the adoptive transfer of Aza-iTreg into the ApoE^−/−^ mice, a significant reduction was observed in both the mRNA and the protein expression levels of the Dnmts (Dnmt1, Dnmt3a, and Dnmt3b) in CD4^+^ T cells from the spleen of the ApoE^−/−^ mice that were administered a WD for eight and 12 weeks. This is shown in Fig. 3A–G.

Considering that Foxp3 serves not only as a reliable marker for CD4^+^ CD25^+^ Tregs, but also plays a pivotal role in their development and function, we investigated the impact of Aza-iTreg transfer on the expression of Foxp3 mRNA. Compared with the Tn cell transfer and the control groups, Aza-iTreg transfer led to a reduction in Foxp3-TSDR methylation levels in the CD4^+^ T cells of mice after being fed the WD for eight and 12 weeks (Fig. 3H, I). Treg transfer also resulted in decreased Foxp3 methylation levels in the CD4^+^ T cells of the eight-week WD group, compared with those in the Tn transfer and the control groups. Similarly, after 12 weeks of the WD, Treg transfer reduced Foxp3-TSDR methylation levels in the CD4^+^ T cells to a greater extent than Tn cell transfer, but there was no difference compared with those of the control group (Fig. 3H, I). The level of Foxp3 methylation in the CD4^+^ T cells following Aza-iTreg transfer was comparable to that following Treg transfer, and was even lower in the Aza-iTreg transfer group.

A decline in the methylation levels of Foxp3-TSDR was evident upon Aza-iTreg transfer and Treg transfer. This reduction in methylation coincided with heightened levels of expression of Foxp3 mRNA (Fig. 3J) and protein (Fig. 3K, L) within the CD4^+^ T cells isolated from the spleen of the ApoE^−/−^ mice. In comparison with the Tn cell transfer and the control groups of mice at the eight-week WD stage, both Aza-iTreg transfer and Treg transfer resulted in augmented expressions of Foxp3 mRNA and protein in the CD4^+^ T cells derived from the spleen of ApoE^−/−^ mice. Furthermore, in contrast with the Tn cell transfer and the control groups after 12 weeks of the WD, both Aza-iTreg transfer and Treg transfer significantly upregulated the levels of Foxp3 mRNA and protein expression within the CD4^+^ T cells from the spleen. The levels of Foxp3 mRNA and protein expressions subsequent to Aza-iTreg transfer were comparable to those observed in case of Treg transfer in the spleen of the ApoE^−/−^ mice. Collectively, these findings suggest that the demethylation of Foxp3-TSDR and the augmentation of Foxp3 expression manifest in the CD4^+^ T cells of ApoE^−/−^ mice after Aza-iTreg transfer.

### Aza induces naive CD4^+^ T cells to Tregs by Dnmt1-mediated Foxp3-TSDR demethylation and upregulation of Foxp3 expression

**Fig. 4:**
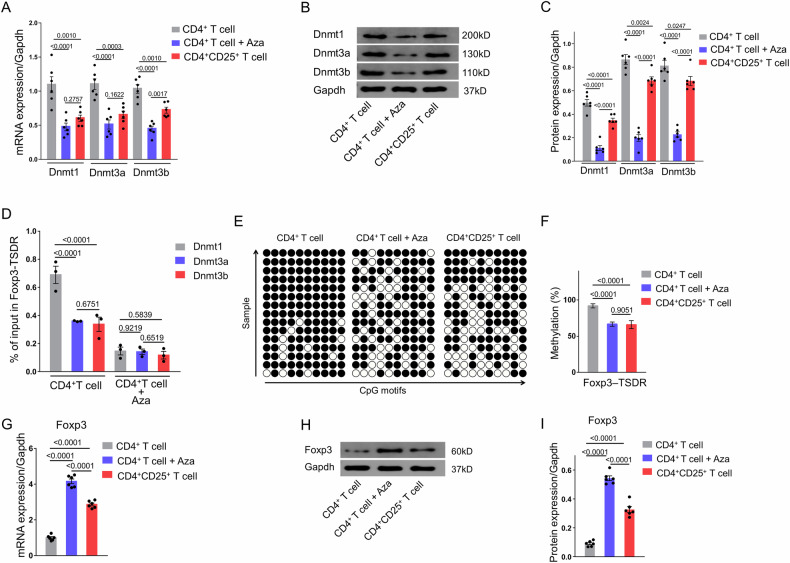
Aza induces the differentiation of naive CD4^+^ T cells into Tregs by mediating the demethylation of Foxp3-TSDR through Dnmt1 and upregulating Foxp3 expression. **A** mRNA expressions of Dnmt1, Dnmt3a, and Dnmt3b in isolated CD4^+^T cells and CD4^+^CD25^+^ T cells (*n* = 6). **B** Results of Western blot of Dnmt1, Dnmt3a, and Dnmt3b in isolated CD4^+^T cells and CD4^+^CD25^+^ T cells. **C** Protein levels of Dnmt1, Dnmt3a, and Dnmt3b in isolated CD4^+^T cells and CD4^+^CD25^+^ T cells (*n* = 6). **D** CHIP assay for Dnmt1, Dnmt3a, and Dnmt3b enrichment in the TSDR region of the Foxp3 locus in the control CD4^+^ T cells and the Aza-CD4^+^ T cells 48 h after induction (*n* = 3). **E** Methylation status of ten individual CpG motifs of the Foxp3-TSDR represented by white (demethylation) and black (methylation) circles. Five repetitions of sequencing per sample. **F** Results of statistical analysis of Foxp3-TSDR methylation in isolated CD4^+^T cells and CD4^+^CD25^+^ T cells (n = 3, repeated five times). **G** mRNA expressions of Foxp3 in isolated CD4^+^T cells and CD4^+^CD25^+^ T cells (*n* = 6). **H** Western blot results of Foxp3 in isolated CD4^+^T cells and CD4^+^CD25^+^ T cells. **I** Protein levels of Foxp3 in isolated CD4^+^T cells and CD4^+^CD25^+^ T cells (*n* = 6). The data are presented as the mean ± SEM. The values on the horizontal lines in the figure are the *P* values between the two groups. Abbreviations: Aza 5-azacytidine, Dnmt DNA methyltransferase, Foxp3 Forkhead box P3, Treg regulatory T cell, TSDR Treg-specific demethylated region.

To elucidate the mechanism through which Aza induces the differentiation of naive CD4^+^ T cells into regulatory Tregs, we isolated CD4^+^ T cells from the spleens of the C57BL/6J mice and subjected them to Aza stimulation. A comparative analysis with untreated CD4^+^ T cells revealed a significant reduction in the levels of expression of mRNA and protein of Dnmt1, Dnmt3a, and Dnmt3b following Aza induction (Fig. 4A–C). Notably, the Aza-treated group exhibited even lower Dnmt expression levels than the CD4^+^ CD25^+^ T cell group. To ascertain the primary Dnmts targeted by Aza for inhibiting Foxp3 methylation, a ChIP assay was conducted to assess the enrichment of Dnmt1, Dnmt3a, and Dnmt3b at the TSDR of the Foxp3 locus in the control CD4^+^ T cells and the Aza-treated CD4^+^ T cells after 48 h of induction (Fig. S5). The results presented in Fig. 4D show a significant enrichment in Dnmt1 at the Foxp3 locus in the CD4^+^ T cells, and this notably decreased following Aza induction.

A discernible decrease in Dnmt1 expression was observed upon Aza stimulation, and corresponded to a significant reduction in the methylation level of the Foxp3-TSDR region (Fig. 4E, F). The methylation levels of Foxp3-TSDR in the Aza induction group were equivalent to those of the CD4^+^ CD25^+^ T cell group (Fig. 4E, F). Likewise, following Aza induction and the subsequent demethylation of Foxp3, a noteworthy increase was noted in both the levels of expression of mRNA and protein of Foxp3, surpassing those observed in the CD4^+^ CD25^+^ T cell group (Fig. 4G–I).

In addition, Aza induction resulted in a significant upregulation of TGF-β and IL-10 concentrations within the CD4^+^ T cells (Figs. S6A and S6B). Conversely, the levels of IL-1β and IFN-γ were significantly reduced after Aza induction (Figs. S6C and S6D). The reduction in inflammatory factors was associated with the increase in Foxp3 expression.

In summary, these findings substantiate that azacitidine induces the differentiation of naive CD4^+^ T cells into Tregs primarily through Dnmt1-mediated Foxp3 demethylation and the subsequent upregulation of Foxp3 expression.

### Overexpression of Dnmt1 attenuates Aza-induced Foxp3-TSDR demethylation and upregulation of Foxp3 expression of CD4^+^ T cells

**Fig. 5:**
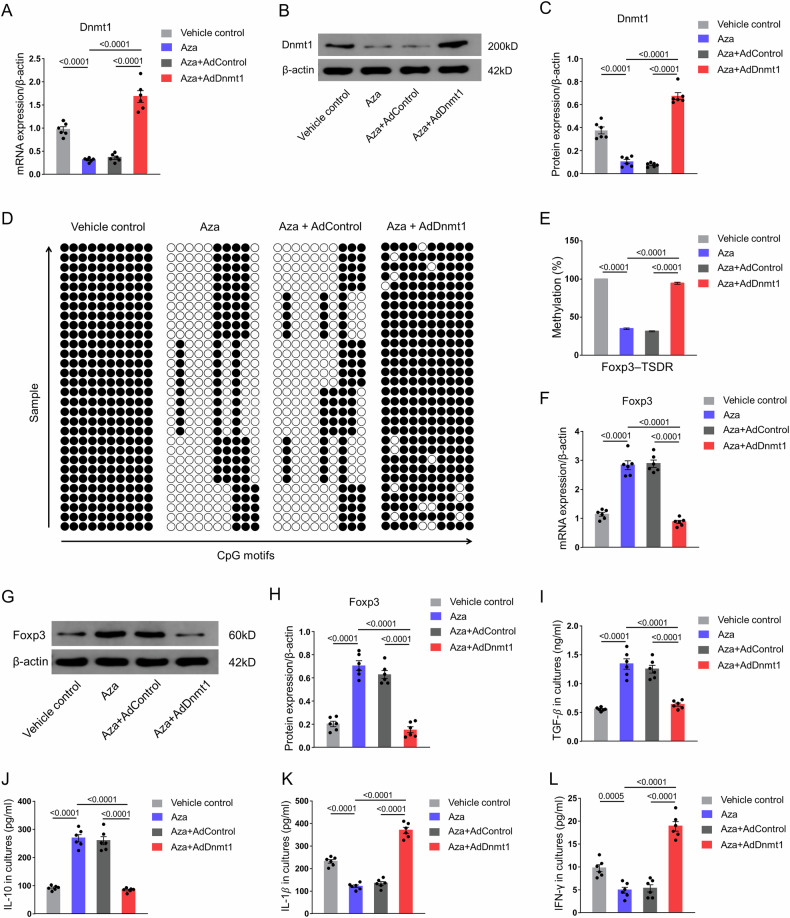
Dnmt1 overexpression attenuates the Aza-induced demethylation of Foxp3-TSDR and upregulation of Foxp3 expression in CD4^+^ T cells. **A** Levels of mRNA expression of Dnmt1 in isolated CD4^+^ T cells (*n* = 6). **B** Western blot results of Dnmt1 in isolated CD4^+^ T cells. **C** Protein level of Dnmt1 in isolated CD4^+^ T cells (*n* = 6). **D** Methylation status of ten individual CpG motifs of the Foxp3-TSDR, represented by white (demethylation) and black (methylation) circles. Five repetitions of sequencing per sample. **E** Results of the statistical analysis of Foxp3-TSDR methylation in isolated CD4^+^ T cells (*n* = 6, repeated five times). **F** Levels of mRNA expression of Foxp3 in isolated CD4^+^ T cells (*n* = 6). **G** Western blot results of Foxp3 in isolated CD4^+^ T cells. **H** Protein level of Foxp3 in isolated CD4^+^ T cells (*n* = 6). **I** Concentrations of TGF-β in isolated CD4^+^ T cells (*n* = 6). **J** Concentrations of IL-10 in isolated CD4^+^ T cells (*n* = 6). **K** Concentrations of IL-1β in isolated CD4^+^ T cells (*n* = 6). **L** Concentrations of IFN-γ in isolated CD4^+^ T cells (*n* = 6). The data are presented as the mean ± SEM. The values on the horizontal lines in the figure are the *P* values between the two groups. Abbreviations: Aza 5-azacytidine, Dnmt DNA methyltransferase, Foxp3 Forkhead box P3, Treg regulatory T cell, TSDR Treg-specific demethylated region.

We subsequently investigated whether the overexpression of mouse Dnmt1 through the adenovirus could mitigate the expression of Foxp3 in the CD4^+^ T cells. Cells infected with AdDnmt1 showed increased Dnmt1 expression (Fig. S2C–E). Moreover, the overexpression of Dnmt1 attenuated the decrease in Dnmt1 expression caused by Aza induction (Fig. 5A–C). In comparison with Aza induction, Dnmt1 overexpression significantly increased the methylation level of Foxp3-TSDR (Fig. 5D and E), and reduced the expression of Foxp3 (Fig. 5F–H). Further, increasing Dnmt1 resulted in a substantial reduction in the concentrations of TGF-β and IL-10, and a significant increase in the concentrations of IL-1β and IFN-γ in isolated CD4^+^ T cells (Fig. 5I–L). In addition, a Dnmt1-selective inhibitor, GSK3685032, was used to demonstrate that inhibition of Dnmt1 promotes the differentiation of CD4^+^ T cells into Tregs (Fig. S7). These findings collectively provide further evidence that azacitidine induces the differentiation of naive CD4^+^ T cells into Tregs by inhibiting Dnmt1.

## Discussion

**Fig. 6:**
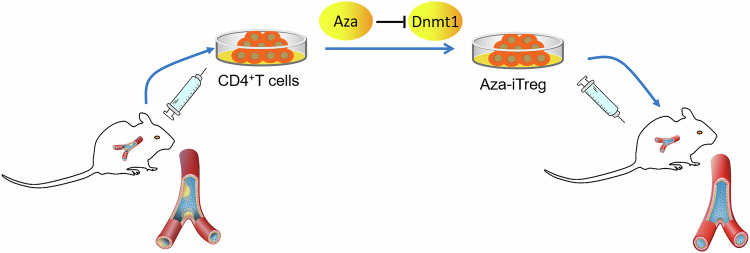
Schematic of the therapeutic role of Aza in atherosclerosis.

The impact of Aza on atherosclerosis has largely remained unexplored in research. This study investigated whether the transfer of naive CD4^+^ T cells, induced to differentiate into Tregs by using Aza in vitro, could suppress atherosclerosis in mice. Moreover, we sought to uncover the mechanisms by which Aza promotes the differentiation of naive CD4^+^ T cells into Tregs. Our findings demonstrated that Aza induced the differentiation of naive CD4^+^ T cells into functional Tregs in vitro. Furthermore, the transfer of Aza-induced Tregs into mice resulted in the suppression of various stages of atherosclerosis. This effect was primarily mediated by the inhibition of Dnmt1, which facilitated the demethylation of Foxp3 and the subsequent upregulation of Foxp3 expression (Fig. 6).

As a methyltransferase inhibitor, Aza is known for its therapeutic applications in various diseases through its modulation of the status of methylation of the relevant genes. For instance, Zhao et al. demonstrated that Aza administration has a protective effect against early renal injury, where this is attributed to its ability to upregulate the expression of klotho [15]. Preconditioning the myocardium with 5-azacytidine has been shown to attenuate injury caused by ischemia and subsequent reperfusion [24, 25]. Aza has also been observed to upregulate the expression of the phosphatase and tensin homolog (PTEN) in smooth muscle cells (SMCs). This upregulation promotes the maintenance of SMC differentiation and contributes to the reduction of pathological vascular remodelling [26]. Previous studies have illustrated that in vitro treatment with Aza has the capacity to convert CD4^+^ conventional T cells into Foxp3-expressing Tregs [18, 27]. Consistent with these previous findings, our results indicated that Aza induces the differentiation of naive CD4^+^ T cells into Tregs by inhibiting Foxp3 methylation and upregulating its expression.

The impact of Aza on disease phenotypes is mediated through its inhibition of Dnmts and promotion of gene expression. However, the specific methyltransferases targeted by Aza can vary depending on the disease and cell type. Keith et al. demonstrated that Aza inhibits Dnmt1 to promote PTEN expression in SMCs, thereby suppressing vascular remodelling [26]. 5-azacytidine inhibits cell proliferation by reversing the abnormally high methylation of the hepaCAM gene through the downregulation of DNMT3A/3B expression in bladder cancer cells [28]. Previous studies have also shown that Aza enhances the expression of specific genes in CD4^+^ T cells by inhibiting Dnmt1 [19, 29]. Consistent with these findings, our observations revealed the strong enrichment of Dnmt1 at the Foxp3 locus, indicating its involvement in the regulation of Foxp3 expression. We also found that the overexpression of Dnmt1 reduced the Aza-induced demethylation of Foxp3-TSDR and the subsequent upregulation of Foxp3 expression in the CD4^+^ T cells. These results align with previous studies, and provide further evidence of the role of Dnmt1 in mediating the effects of Aza on CD4^+^ T cell differentiation into Tregs.

The therapeutic potential of Tregs in atherosclerosis has indeed been explored over the past two decades, as highlighted by seminal studies [10, 30]. These studies demonstrated the protective effects of Tregs in murine models of atherosclerosis, with both Tr1 cells and naturally occurring CD4^+^ CD25^+^ Tregs showing significant atheroprotective properties through immune modulation. While these findings established the foundation for Treg-mediated atherosclerosis treatment, several challenges have remained unresolved, particularly concerning the practical application of these therapies and the mechanisms underlying Treg differentiation and function in this context. Our study advances this field by introducing an innovative approach that leverages the epigenetic modification of naïve CD4^+^ T cells using Aza to generate functional Tregs. Unlike earlier studies that primarily focused on the therapeutic use of naturally occurring or antigen-specific Tregs, our research emphasizes the potential of Aza as a tool to induce Treg differentiation in vitro. This approach not only broadens the scope of Treg-based therapies by providing a method to generate Tregs from naïve CD4^+^ T cells but also offers a deeper understanding of the molecular mechanisms involved, particularly the role of Dnmt1 inhibition and subsequent Foxp3 upregulation through demethylation.

The number of Tregs is limited, and allogeneic Treg infusion is prone to induce immune rejection [7]. Consequently, obtaining a sufficient number of regulatory T cells while avoiding immunological rejection presents a significant challenge. Previous studies have used immunotherapy to stimulate mice to produce more Tregs by exogenously administering antigens to suppress atherosclerosis [31, 32]. Nevertheless, Tregs and inflammatory T cells often share activation pathways, such as the T cell receptor (TCR) signalling pathway, and there is substantial variability in the strength of the TCR signals. Exogenous antigen activation induces the generation of Tregs while potentially activating and amplifying inflammatory T cells [33, 34]. In our research, we made the noteworthy discovery that CD4^+^ T cells exhibited stable Treg functionality when induced with Aza. This is particularly important because iTregs can be derived from naive CD4^+^ T cells [10], and can be easily manipulated outside the body. The specificity of iTregs derived from autologous CD4^+^ T cells plays a crucial role in circumventing immunological rejection, which is a major advantage. Furthermore, while Tregs are relatively scarce, constituting only 5%–10% of CD4^+^ T cells [7], the latter themselves are more abundant. Through the in vitro induction of CD4^+^ T cells with Aza, we were able to generate a substantial number of Tregs, thus addressing the issue of Treg scarcity. Consequently, Aza-induced autologous CD4^+^ T cells offer a promising solution for alleviating atherosclerosis by ensuring a sufficient number of Tregs and minimising the risk of immune rejection. Our findings highlight the potential of using Aza-iTregs as a viable cellular therapeutic approach in models of atherosclerosis.

Our findings have the potential to inspire novel approaches in clinical practice. This approach not only provides a robust preclinical model but also paves the way for potential clinical applications. The use of Aza to epigenetically modify autologous CD4^+^ T cells and subsequently reintroduce them into patients offers a promising strategy to enhance Treg numbers and efficacy in treating atherosclerosis, thereby addressing both the limitations of Treg scarcity and the challenges of immune rejection. By focusing on this innovative approach, our findings could inspire new clinical strategies for the treatment of atherosclerosis.

## Conclusions

Our findings demonstrate that iTregs can be epigenetically edited ex vivo by using the Aza-based approach through the inhibition of Dnmt1, and that Aza-iTregs can suppress atherosclerosis in vivo. These findings suggest that using a substantial number of Aza-induced iTreg cells for cellular therapy offers great promise for the treatment of atherosclerosis.

## Supplementary information

Supplementary materials

Full and uncropped WB images

## Data Availability

The supporting data for the findings of this study can be obtained from the corresponding author upon reasonable request.
