# Supplementary material for: Epigenetic modification of CD4+ T cells into Tregs by 5-azacytidine as cellular therapeutic for atherosclerosis treatment
Source: Cell Death Dis. 2024 Sep 20;15(9):689. doi: 10.1038/s41419-024-07086-7 (PMC11415506; doi:10.1038/s41419-024-07086-7)
Supplement: Supplementary file 1 — Supplementary materials [file 41419_2024_7086_MOESM1_ESM.docx]

**Supplemental Material**

**Contents**

**Ⅰ Supplemental Figures**

**Figure S1:** The diagram of animal experiments.

**Figure S2:** Construction, and transduction efficiency of Ad-ZsGreen-mDnmt1.

**Figure S3:** Cell viability of isolated CD4^+^T cells and CD4^+^CD25^+^ T cells prior to injection through the tail vein by trypan blue staining.

**Figure S4:** Adoptive transfer of Aza-iTreg reduced whole aorta lesions in ApoE^−/−^ mice.

**Figure S5:** CHIP assay for Dnmt1, Dnmt3a, and Dnmt3b enrichment at the TSDR region of Foxp3 locus.

**Figure S6:** Aza induction significantly inhibited inflammatory factor levels of isolated CD4^+^ T cells.

**Figure S7:** Dnmt1 overexpression attenuates the GSK3685032-induced demethylation of Foxp3-TSDR and upregulation of Foxp3 expression in CD4^+^ T cells.

**Ⅱ Supplemental Tables**

**Table S1:** Sequences of Foxp3-TSDR for bisulfite sequencing.

**Table S2:** Primers sequence of quantitative RT-PCR.

**Table S3:** Sequences of mouse Foxp3-TSDR for ChIP.

**Table S4:** Sequences of mouse mDnmt1 for Ad-ZsGreen-mDnmt


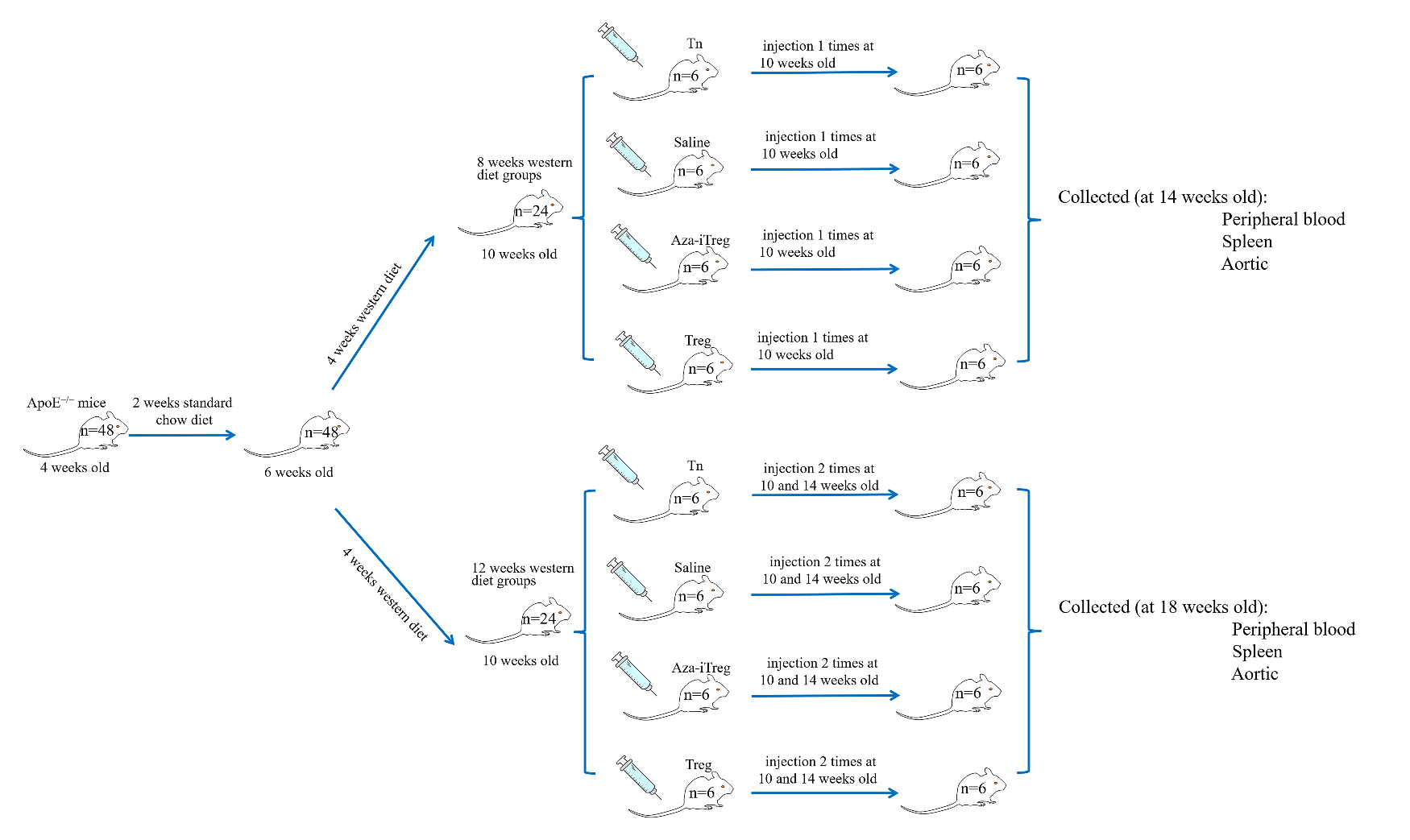


**Figure S1:** The diagram of animal experiments. Abbreviations: Aza, Azacytidine.

Abbreviations: Aza, 5-Azacytidine; Tn, Naive CD4^+^ T cell; Treg, regulatory T cell.


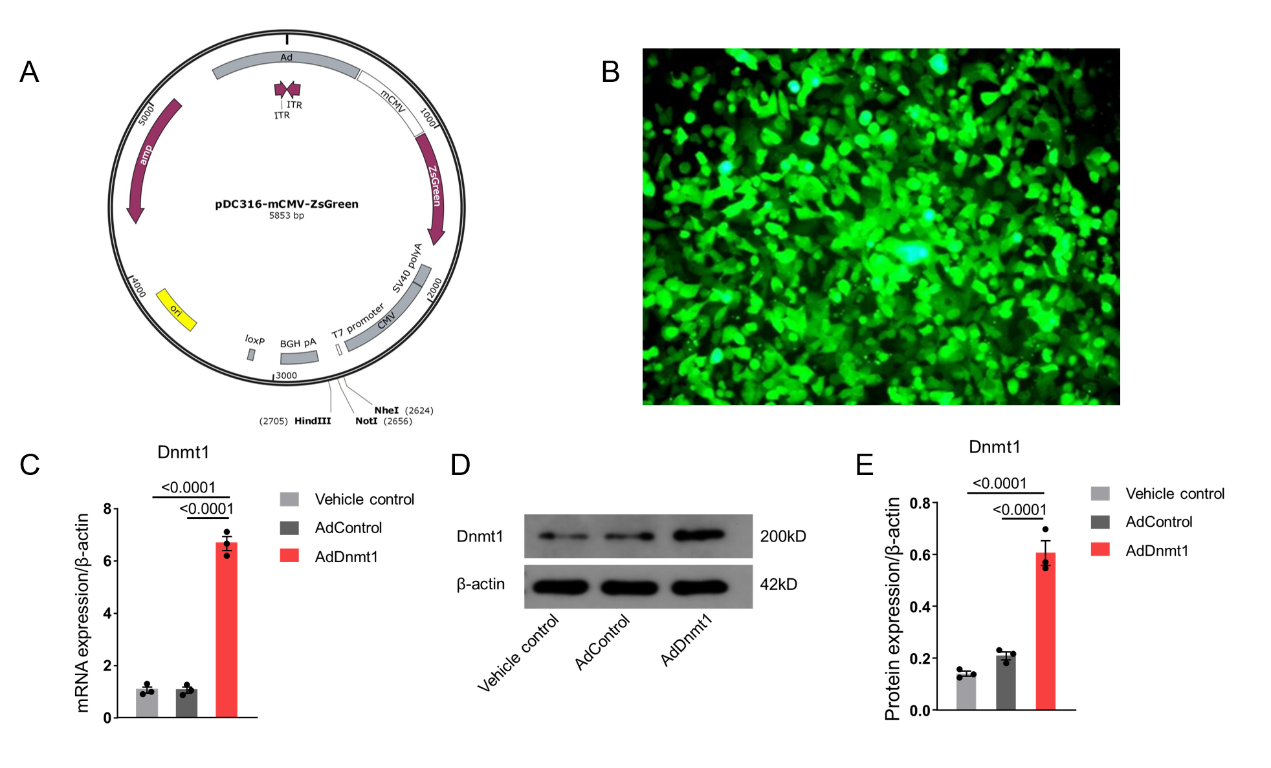


**Figure S2:** Construction, and transduction efficiency of Ad-ZsGreen-mDnmt1. **(A)** Structure of Ad-ZsGreen-mDnmt1. **(B)** Transduction of viral vectors carrying Ad-ZsGreen-mDnmt1 into isolated CD4^+^ T cells. **(C)** The mRNA level of Dnmt1 of isolated CD4^+^T cells transfected by viral vector carrying AdDnmt1 (n = 3). **(D)** and **(E)** The protein level of Dnmt1 of isolated CD4^+^T cells transfected by viral vector carrying AdDnmt1 (n = 3).

Data are presented as the mean ± SEM. The values on the horizontal lines in the figure are the P values between the two groups.

Abbreviations: Dnmt1, DNA methyltransferase 1.

**
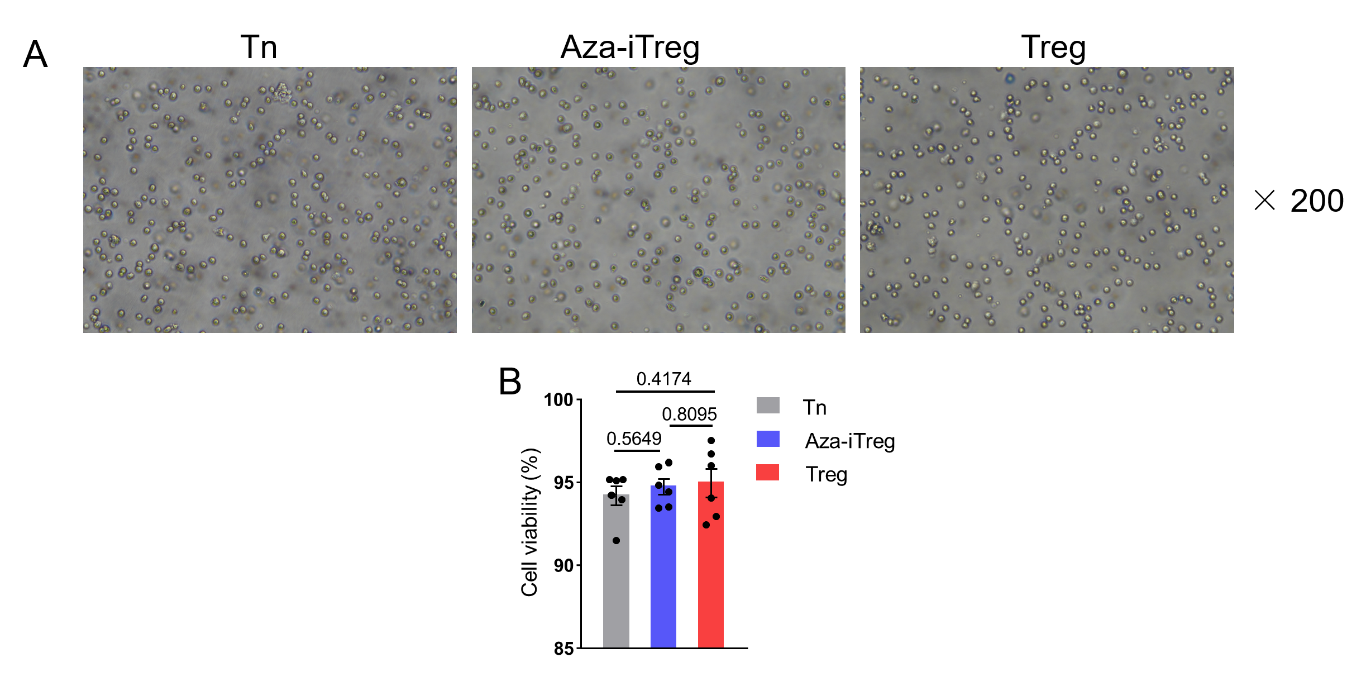
**

**Figure S3:** Cell viability of isolated CD4^+^T cells and CD4^+^CD25^+^ T cells prior to injection through the tail vein by trypan blue staining. **(A)** Cell viability of isolated CD4^+^T cells, magnification ×200. **(B)** Results of the statistical analysis of cell viability among three groups (n = 6).

Data are presented as the mean ± SEM. The values on the horizontal lines in the figure are the P values between the two groups.

Abbreviations: Aza, 5-Azacytidine; Tn, Naive CD4^+^ T cell; Treg, regulatory T cell.

**
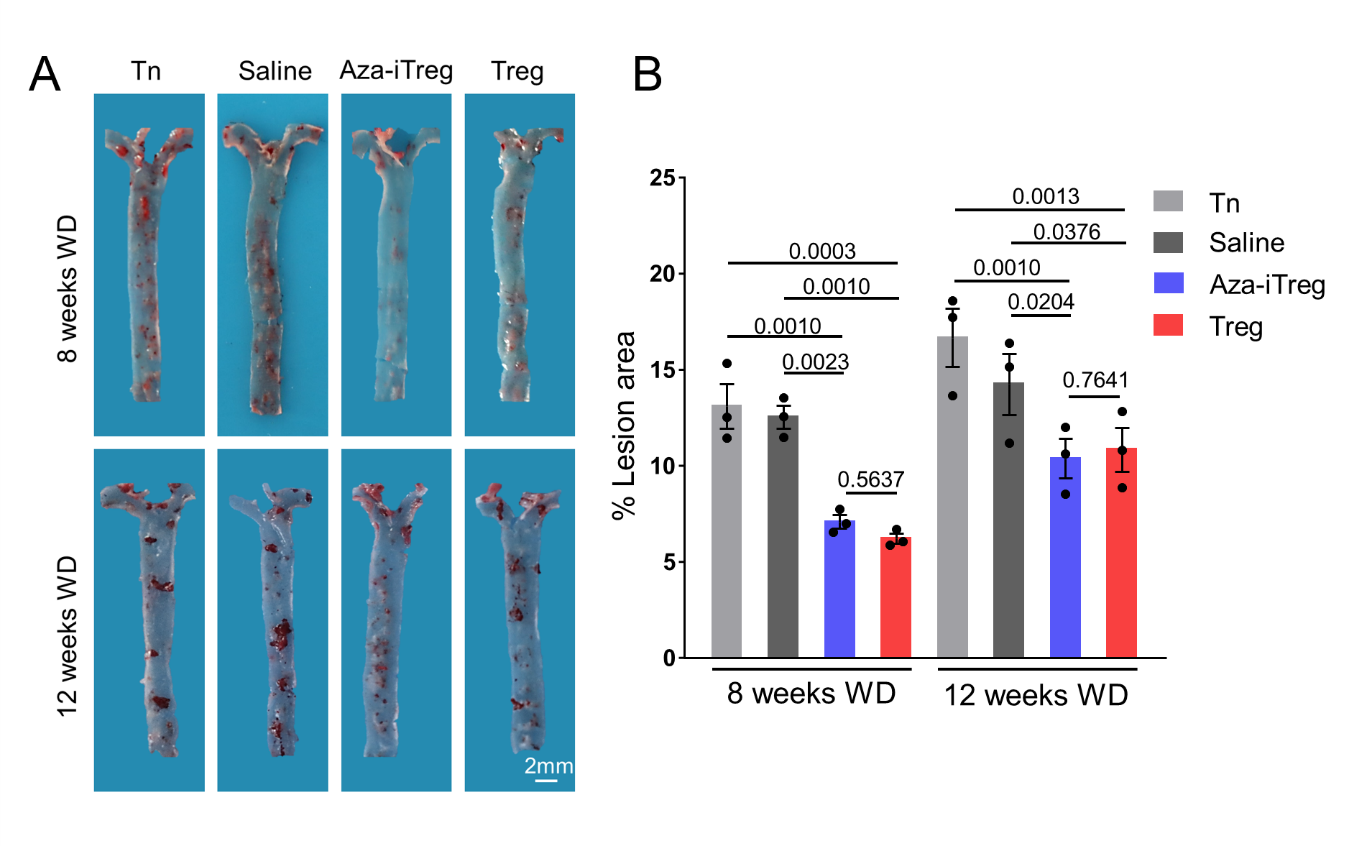
**

**Figure S4:** Adoptive transfer of Aza-iTreg reduced whole aorta lesions in ApoE^−/−^ mice. **(A)** The fraction area of lesion was measured by Oil Red O staining. **(B)** Quantitative analysis of whole aorta lesions area (n = 3).

Data are presented as the mean ± SEM. The values on the horizontal lines in the figure are the P values between the two groups.

Abbreviations: Aza, 5-Azacytidine; Tn, Naive CD4^+^ T cell; Treg, regulatory T cell; WD, western diet.

**
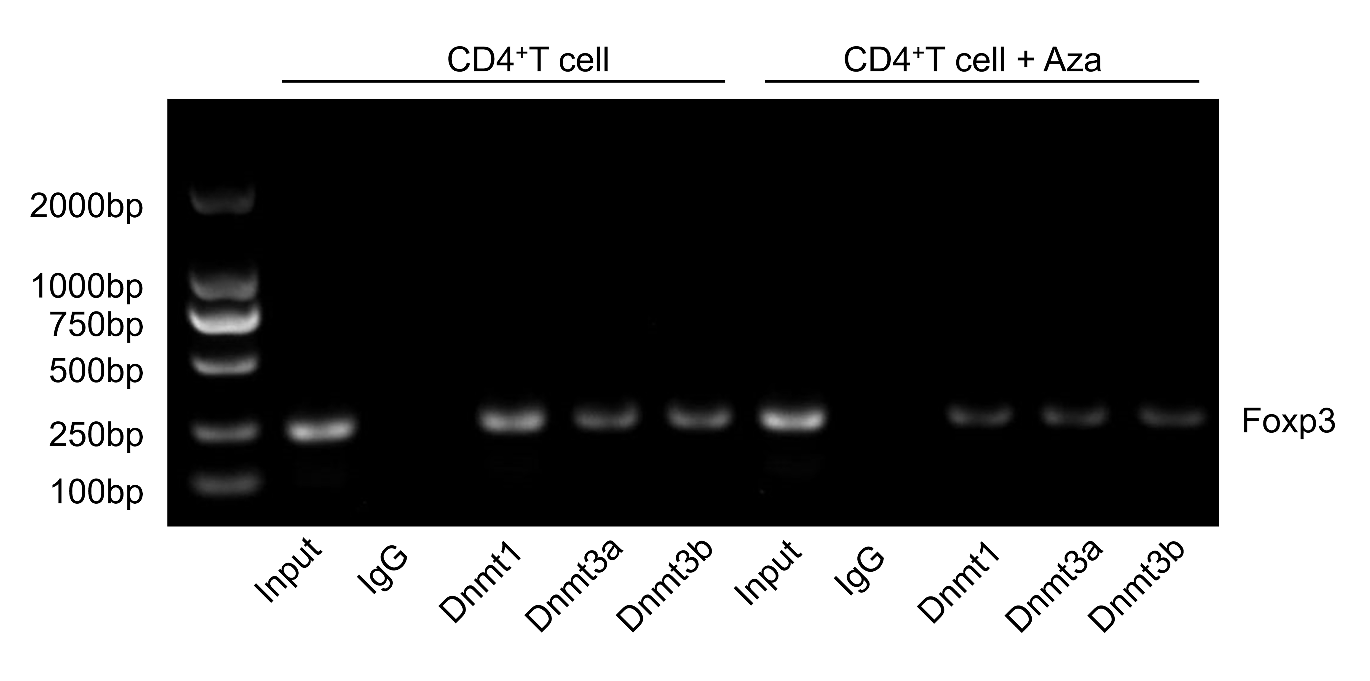
**

**Figure S5:** CHIP assay for Dnmt1, Dnmt3a, and Dnmt3b enrichment at the TSDR region of Foxp3 locus in control CD4^+^ T cells and AZA-CD4^+^ T cells 48 h after induction.

Abbreviations: Aza, 5-Azacytidine; ChIP, Chromatin immunoprecipitation; Foxp3, forkhead box P3; TSDR, Treg-specific demethylated region.

**
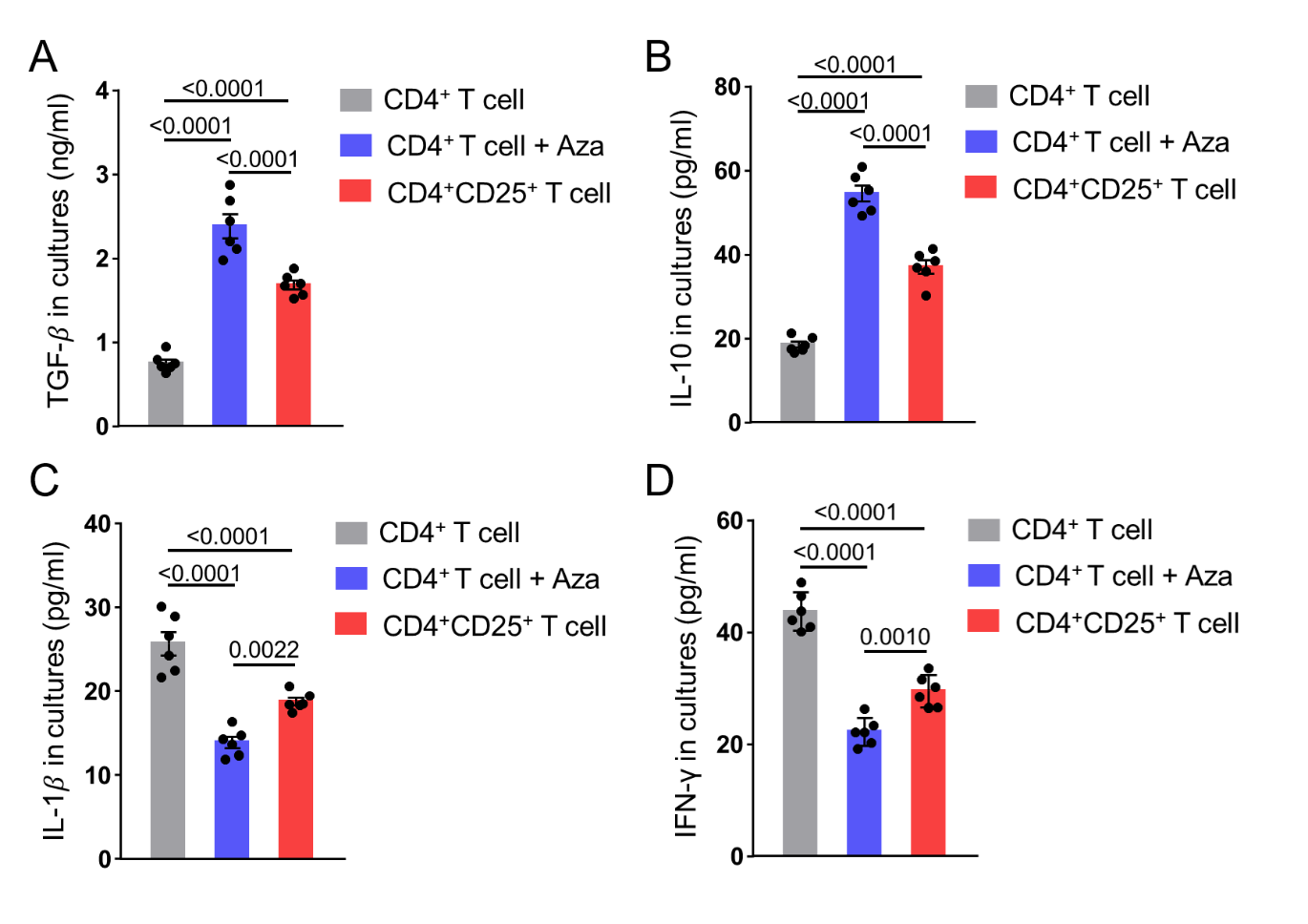
**

**Figure S6:** Aza induction significantly inhibited inflammatory factor levels of isolated CD4^+^ T cells. **(A)** The concentrations of TGF-β in isolated CD4^+^T cells and CD4^+^CD25^+^ T cells (n = 6). **(B)** The concentrations of IL-10 in isolated CD4^+^T cells and CD4^+^CD25^+^ T cells (n = 6). **(C)** The concentrations of IL-1β in isolated CD4^+^T cells and CD4^+^CD25^+^ T cells (n = 6). **(D)** The concentrations of IFN-γ in isolated CD4^+^T cells and CD4^+^CD25^+^ T cells (n = 6).

Data are presented as the mean ± SEM. The values on the horizontal lines in the figure are the P values between the two groups.

Abbreviations: Aza, 5-Azacytidine.


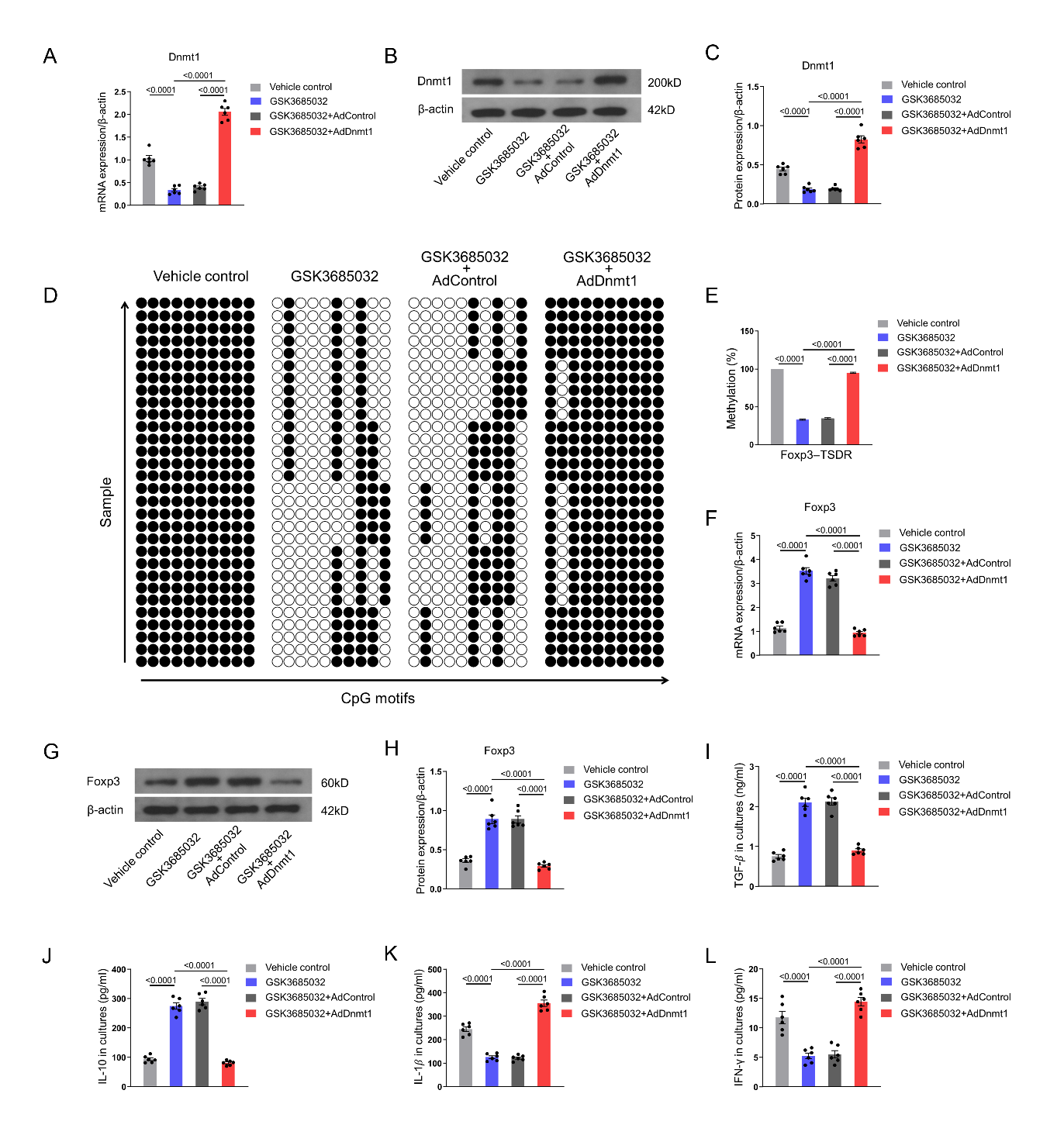


**Figure S7:** Dnmt1 overexpression attenuates the GSK3685032-induced demethylation of Foxp3-TSDR and upregulation of Foxp3 expression in CD4^+^ T cells.

(A) Levels of mRNA expression of Dnmt1 in isolated CD4^+^ T cells (n = 6). (B) Western blot results of Dnmt1 in isolated CD4^+^ T cells. (C) Protein level of Dnmt1 in isolated CD4^+^ T cells (n = 6). (D) Methylation status of ten individual CpG motifs of the Foxp3-TSDR, represented by white (demethylation) and black (methylation) circles. Five repetitions of sequencing per sample. (E) Results of the statistical analysis of Foxp3-TSDR methylation in isolated CD4^+^ T cells (n = 6, repeated five times). (F) Levels of mRNA expression of Foxp3 in isolated CD4^+^ T cells (n = 6). (G) Western blot results of Foxp3 in isolated CD4^+^ T cells. (H) Protein level of Foxp3 in isolated CD4^+^ T cells (n = 6). (I) Concentrations of TGF-β in isolated CD4^+^ T cells (n = 6). (J) Concentrations of IL-10 in isolated CD4^+^ T cells (n = 6). (K) Concentrations of IL-1β in isolated CD4^+^ T cells (n = 6). (L) Concentrations of IFN-γ in isolated CD4^+^ T cells (n = 6).

The data are presented as the mean ± SEM. The values on the horizontal lines in the figure are the P values between the two groups. The CD4^+^ T cells were stimulated with a DNA methyltransferase inhibitor (GSK3685032, Selleck) at a concentration of 5 μM and 48 hours.

Abbreviations: Dnmt, DNA methyltransferase; Foxp3, Forkhead box P3; Treg, regulatory T cell; TSDR, Treg-specific demethylated region.

**Table S1 Sequences of Foxp3-TSDR for bisulfite sequencing**

| Assay type | Primer | Sequence |
| --- | --- | --- |
| Mouse methylation specific qPCR | Forward | 5′-TTGGGTTTTTTTGGTATTTAAGAAA-3′ |
|  | Reverse | 5′-CCCTATTATCACAACCTAAACTTAACC-3′ |
| Bisulfite sequencing of mouse *Foxp3*-TSDR |  | TTGGGTTTTTTTGGTATTTAAGAAAGATAGAATCGATAGAATTTGGGTTTTGTATGGTAGTTAGATGGACGTTATTTATTATATTCGTTAGCATTTATATTATTTTATTTGGGTTTATTCGGTTATAGGATAGATTAGTTATTTTTCGGAACGAAATTTGTGGGGTAGATTATTTGTTTTTTTTTTTTTTTTTTTGTTGTCGATGAAGTTTAATGTATTCGGTCGTTATGACGTTAATGGTAGAAAAATTTGGTTAAGTTTAGGTTGTGATAATAGGG |

CG: CpG site of bisulfite sequencing.

Abbreviations: Foxp3, Forkhead box P3; TSDR, Treg-specific demethylated region.

**Table S2 Primers sequence of quantitative RT-PCR**

| Name | Primer | Sequence | Size |
| --- | --- | --- | --- |
| Mouse Foxp3 | Forward | 5‘-CCTTTCACCTATGCCACCCTTATCC-3’ | 198bp |
|  | Reverse | 5‘- TGCTCCCTTCTCGCTCTCCACT-3’ |  |
| Mouse Dnmt1 | Forward | 5‘- CGGCTCAAAGACTTGGAAAG -3’ | 163bp |
|  | Reverse | 5‘- TAGCCAGGTAGCCTTCCTCA -3’ |  |
| Mouse Dnmt3a | Forward | 5‘- GCGATTTCTTGAGTCTAACCC -3’ | 120bp |
|  | Reverse | 5‘- TTCACAGTGGATGCCAAAGG -3’ |  |
| Mouse Dnmt3b | Forward | 5‘-TTGGTGCTCAAGGAGTTGGGTATTA-3’ | 217bp |
|  | Reverse | 5‘-GGGCAGGATTGACGTTAGAGAGA-3’ |  |
| Mouse Gapdh | Forward | 5‘-ATGGGTGTGAACCACGAGA-3’ | 229bp |
|  | Reverse | 5‘-CAGGGATGATGTTCTGGGCA-3’ |  |
| Mouse β-actin | Forward | 5‘-CACGATGGAGGGGCCGGACTCATC-3’ | 240bp |
|  | Reverse | 5‘-TAAAGACCTCTATGCCAACACAGT-3’ |  |

Abbreviations: Dnmt1, DNA methyltransferases 1; Dnmt3a, DNA methyltransferases 3a; Dnmt3b, DNA methyltransferases 3b; Foxp3, Forkhead box P3; Gapdh, glyceraldehyde-3-phosphate dehydrogenase; TSDR, Treg-specific demethylated region.

**Table S3 Sequences of mouse Foxp3-TSDR for ChIP**

| Assay type | Primer | Sequence |
| --- | --- | --- |
| Mouse foxp3 primers sequence of PCR for ChIP | Forward | 5′- AGAACTTGGGTTTTGCATGG -3′ |
|  | Reverse | 5′- GCCAGATTTTTCTGCCATTG -3′ |
| Sequences of mouse Foxp3-TSDR for ChIP |  | TTGGGCCCCTCTGGCATCCAAGAAAGACAGAATCGATAGAACTTGGGTTTTGCATGGTAGCCAGATGGACGTCACCTACCACATCCGCTAGCACCCACATCACCCTACCTGGGCCTATCCGGCTACAGGATAGACTAGCCACTTCTCGGAACGAAACCTGTGGGGTAGATTATCTGCCCCCTTCTCTTCCTCCTTGTTGCCGATGAAGCCCAATGCATCCGGCCGCCATGACGTCAATGGCAGAAAAATCTGGCCAAGTTCAGGTTGTGACAACAGGG |

Abbreviations: ChIP, chromatin immunoprecipitation; Foxp3, Forkhead box P3; TSDR, Treg-specific demethylated region.

**Table S4 Sequences of mouse mDnmt1 for Ad-ZsGreen-mDnmt1**

| Assay type |  | Sequence |
| --- | --- | --- |
| mDnmt1-NotI-F: | | 5′-TCGAGCGGCCGCCACCATGCCAGCGCGAAC  AGCTCCAGCCCGAG-3′ |
| mDnmt1-HindIII-R: | | 5′-CTTAAGCTTCTAGTCCTTGGTAGCAGCCTCC  TCTTTT-3′ |
| Sequences of mouse mDnmt1 (4881bp) |  | GCGGCCGCCACCATGCCAGCGCGAACAGCTCCAGCCCGAGTGCCTGCGCTTGCCTCCCCGGCAGGCTCGCTCCCGGACCATGTCCGCAGGCGGCTCAAAGACTTGGAAAGAGATGGCTTAACAGAAAAGGAGTGTGTGAGGGAGAAATTAAACTTACTGCATGAATTCCTGCAAACAGAAATAAAAAGCCAGTTGTGTGACTTGGAAACCAAATTACATAAAGAGGAATTATCTGAGGAAGGCTACCTGGCTAAAGTCAAGTCCCTCTTAAATAAGGATTTGTCCTTGGAGAACGGAACACACACTCTCACTCAAAAAGCCAACGGTTGTCCCGCCAACGGGAGCCGGCCAACCTGGAGAGCAGAAATGGCAGACTCAAATAGATCCCCAAGATCCAGGCCCAAGCCTCGGGGACCCAGGAGAAGCAAGTCGGACAGTGACACCCTTTCAGTTGAAACTTCACCTAGTTCCGTGGCTACGAGGAGAACCACCAGGCAGACCACCATCACGGCTCACTTCACGAAGGGCCCCACTAAACGGAAACCCAAGGAAGAGTCGGAAGAGGGGAACTCGGCTGAGTCGGCTGCAGAGGAGAGAGACCAGGATAAGAAACGCAGAGTTGTAGACACAGAGAGTGGTGCTGCAGCTGCTGTGGAGAAACTGGAAGAGGTAACAGCGGGAACCCAGCTGGGTCCGGAAGAGCCATGTGAACAGGAAGATGACAACAGGAGTCTTCGACGTCACACCAGAGAGCTATCATTGAGGCGGAAATCAAAGGAGGATCCAGACAGAGAAGCAAGACCGGAAACTCACTTGGACGAGGACGAGGACGGAAAAAAGGATAAAAGAAGTTCCAGACCCAGGAGCCAGCCCAGAGATCCAGCTGCCAAACGGAGACCCAAGGAAGCAGAGCCAGAGCAGGTAGCTCCAGAGACTCCCGAGGACAGAGACGAGGATGAGAGGGAGGAGAAGAGACGAAAAACGACACGTAAAAAACTGGAGTCACACACCGTTCCCGTTCAGAGCAGATCGGAGAGAAAAGCCGCTCAAAGCAAAAGTGTGATCCCGAAGATCAACTCACCAAAGTGCCCCGAGTGTGGCCAGCACCTAGACGACCCTAACCTGAAGTACCAGCAGCACCCTGAGGATGCTGTGGATGAACCCCAGATGTTGACCAGTGAGAAACTGTCCATCTACGACTCCACCTCGACCTGGTTTGATACTTATGAAGATTCTCCCATGCATAGGTTCACTTCCTTCAGTGTGTACTGCAGTCGCGGGCACCTGTGTCCTGTCGACACCGGTCTCATTGAGAAGAATGTAGAGCTCTACTTTTCTGGGTGTGCCAAAGCAATTCATGACGAGAATCCATCTATGGAAGGTGGTATTAATGGCAAAAACCTCGGGCCAATCAATCAGTGGTGGCTCAGTGGCTTTGATGGTGGCGAGAAGGTGCTCATTGGCTTCTCCACTGCATTTGCTGAATACATTTTGATGGAGCCCAGCAAAGAGTATGAGCCAATATTTGGGCTGATGCAGGAGAAAATTTACATCAGCAAGATTGTTGTTGAGTTCCTGCAAAACAATCCTGATGCTGTATATGAAGACCTGATCAATAAGATTGAGACCACTGTTCCTCCTTCTACCATTAATGTGAACCGGTTCACAGAGGACTCCCTCTTACGCCACGCCCAGTTTGTAGTGAGCCAGGTAGAGAGTTACGACGAAGCCAAGGACGATGATGAGACCCCCATCTTCTTGTCTCCCTGTATGAGAGCCCTGATCCATTTGGCTGGTGTCTCCCTGGGACAGAGGCGAGCAACAAGGCGCGTCATGGGTGCTACCAAGGAGAAGGACAAAGCACCCACGAAAGCCACCACCACCAAGCTGGTCTATCAGATCTTTGACACTTTCTTCTCAGAGCAGATTGAGAAGTATGATAAGGAGGACAAGGAGAATGCCATGAAGCGCCGCCGCTGTGGTGTCTGTGAGGTCTGTCAGCAGCCTGAGTGTGGGAAGTGCAAGGCGTGCAAAGATATGGTGAAGTTTGGTGGCACTGGACGGAGTAAGCAGGCTTGCCTCAAGAGGAGGTGTCCTAACTTGGCGGTGAAGGAGGCAGACGACGATGAAGAGGCTGATGATGATGTGTCAGAGATGCCATCACCCAAAAAGCTGCATCAGGGGAAGAAGAAGAAGCAGAACAAGGACCGCATCTCCTGGCTTGGGCAGCCTATGAAGATTGAAGAGAATAGAACTTACTATCAGAAGGTGAGCATCGATGAGGAGATGCTAGAGGTGGGCGACTGCGTCTCGGTCATTCCAGATGATTCCTCCAAACCACTCTATCTAGCCAGGGTCACAGCTCTGTGGGAAGACAAAAATGGTCAGATGATGTTCCATGCGCACTGGTTCTGCGCTGGGACAGACACAGTCCTGGGAGCCACCTCCGACCCCCTGGAACTGTTCCTGGTGGGCGAGTGCGAAAACATGCAGCTTTCCTACATCCACAGCAAGGTCAAGGTCATCTACAAAGCCCCTTCTGAAAACTGGGCCATGGAGGGAGGCACAGACCCTGAGACCACACTGCCTGGGGCTGAGGATGGCAAGACTTACTTCTTCCAGCTCTGGTACAACCAGGAGTACGCAAGGTTTGAATCCCCACCCAAGACCCAGCCGACCGAGGACAACAAGCACAAGTTCTGCCTATCTTGTATCCGGCTGGCTGAGCTGAGACAAAAAGAAATGCCCAAGGTCCTGGAACAAATTGAGGAGGTGGATGGCCGGGTCTACTGCAGTTCCATCACCAAGAATGGTGTTGTCTACCGACTGGGTGACAGTGTGTACCTTCCTCCCGAGGCCTTTACTTTCAACATCAAAGTGGCTAGCCCCGTGAAACGCCCAAAGAAGGATCCTGTGAACGAGACCCTGTACCCTGAGCACTACCGCAAGTATTCTGACTACATCAAGGGGAGCAACCTGGATGCTCCAGAGCCCTATCGCATCGGTCGGATAAAAGAGATCCACTGTGGCAAGAAGAAAGGCAAGGTCAACGAGGCAGACATCAAGCTGAGGCTCTACAAGTTCTACAGGCCTGAGAATACCCACAGGTCCTACAACGGATCCTATCACACTGACATCAACATGCTTTACTGGAGCGACGAGGAAGCTGTGGTGAACTTCAGCGACGTGCAGGGCCGCTGTACCGTGGAGTACGGGGAAGACCTACTTGAGAGCATCCAGGATTATTCACAAGGGGGCCCTGACCGCTTCTACTTCCTCGAGGCCTACAATTCAAAGACCAAGAACTTTGAAGACCCACCAAACCATGCCCGCAGCCCTGGGAACAAAGGGAAAGGGAAGGGGAAAGGGAAGGGGAAGGGGAAGCATCAGGTGTCAGAGCCCAAAGAGCCTGAGGCAGCCATCAAACTGCCCAAGCTCCGGACCCTGGATGTGTTTTCCGGCTGTGGAGGGTTATCGGAAGGATTCCACCAAGCAGGCATCTCGGAAACGCTGTGGGCCATCGAGATGTGGGACCCGGCAGCCCAGGCATTTCGGCTGAACAACCCCGGCACCACAGTGTTCACAGAGGACTGCAACGTGCTTCTTAAGCTGGTCATGGCTGGGGAGGTGACCAACTCTCTGGGCCAAAGGCTGCCACAGAAGGGCGATGTGGAGATGCTGTGTGGTGGGCCACCCTGCCAGGGCTTCAGTGGCATGAACCGCTTCAACTCCCGCACTTACTCCAAGTTCAAAAACTCCCTAGTGGTCTCCTTCCTCAGCTACTGTGACTACTACCGGCCTCGGTTCTTCCTTCTGGAGAACGTCAGGAACTTCGTGTCCTACAGACGCTCCATGGTGCTGAAGCTCACACTGCGCTGCCTGGTCCGCATGGGCTACCAGTGCACCTTTGGTGTGCTCCAGGCTGGACAGTATGGCGTGGCCCAGACACGAAGGAGGGCCATCATCTTGGCTGCAGCCCCAGGAGAAAAGCTGCCTCTGTTCCCAGAGCCTCTGCATGTGTTTGCGCCCCGTGCCTGCCAGCTGAGCGTTGTGGTGGATGACAAGAAGTTTGTTAGCAACATAACGAGGCTGAGCTCGGGGCCCTTCCGAACCATCACCGTGCGAGACACCATGTCTGACCTCCCCGAGATCCAGAATGGAGCCTCGAATTCTGAGATCCCCTACAATGGAGAGCCACTGTCCTGGTTCCAGAGGCAGCTGCGAGGATCACACTACCAGCCCATCCTCAGGGACCATATCTGCAAGGACATGAGCCCACTGGTGGCTGCCCGCATGCGGCACATCCCACTGTTCCCAGGATCAGATTGGCGTGACCTGCCCAACATACAGGTGCGGCTGGGAGATGGCGTCATAGCCCATAAGCTACAGTACACCTTTCATGATGTGAAAAATGGCTACAGCAGCACCGGTGCCCTGCGTGGAGTCTGTTCCTGTGCAGAAGGCAAGGCCTGCGACCCTGAGTCCAGGCAATTCAGCACCCTCATCCCCTGGTGCCTGCCGCACACTGGGAACCGGCACAACCACTGGGCTGGCCTCTACGGGCGTCTGGAGTGGGATGGCTTCTTCAGCACCACTGTCACCAACCCTGAGCCCATGGGCAAGCAGGGTCGGGTGCTCCACCCGGAGCAGCACCGGGTCGTGAGTGTTCGGGAATGTGCCCGCTCCCAGGGCTTTCCAGATAGCTACCGGTTCTTCGGCAACATCCTGGACAGACACCGGCAGGTGGGTAATGCTGTGCCACCACCCCTGGCCAAAGCCATTGGCCTGGAGATTAAGCTCTGCCTGCTGTCCAGTGCTCGGGAGAGCGCATCAGCTGCAGTTAAAGCAAAAGAGGAGGCTGCTACCAAGGACTAGAAGCTT |

Abbreviations: Dnmt1, DNA methyltransferases 1.
