# Supplementary material for: Epigenetic modification of CD4+ T cells into Tregs by 5-azacytidine as cellular therapeutic for atherosclerosis treatment
Source: Cell Death Dis. 2024 Sep 20;15(9):689. doi: 10.1038/s41419-024-07086-7 (PMC11415506; doi:10.1038/s41419-024-07086-7)
Supplement: Supplementary file 2 — Full and uncropped WB images [file 41419_2024_7086_MOESM2_ESM.pdf]

Figure 3G

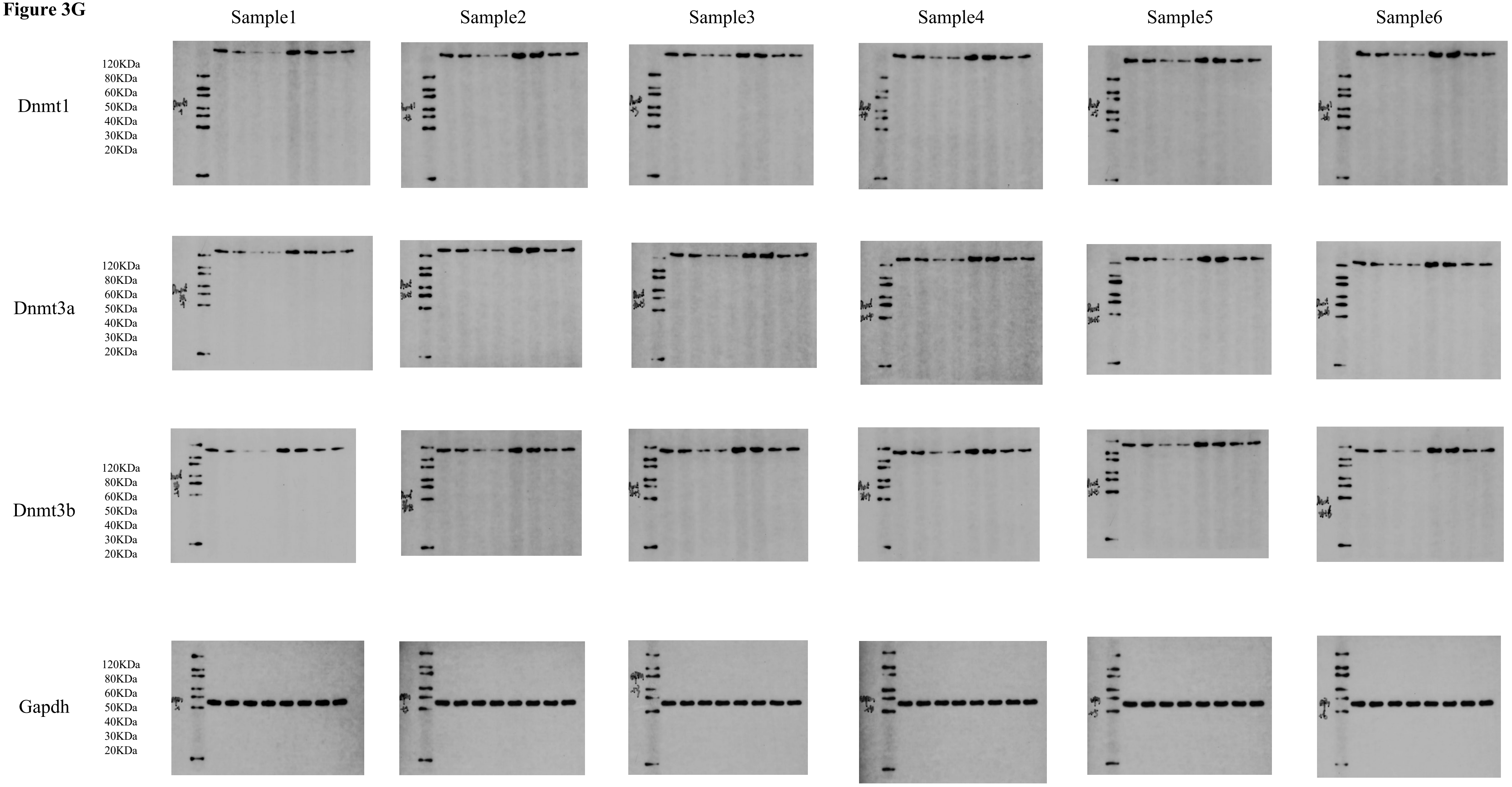
Sample1
Sample2
Sample3
Sample4
Sample5
Sample6
Dnmt1
Dnmt3a
Dnmt3b
Gapdh
120KDa
80KDa
60KDa
50KDa
40KDa
30KDa
20KDa

Figure 3K

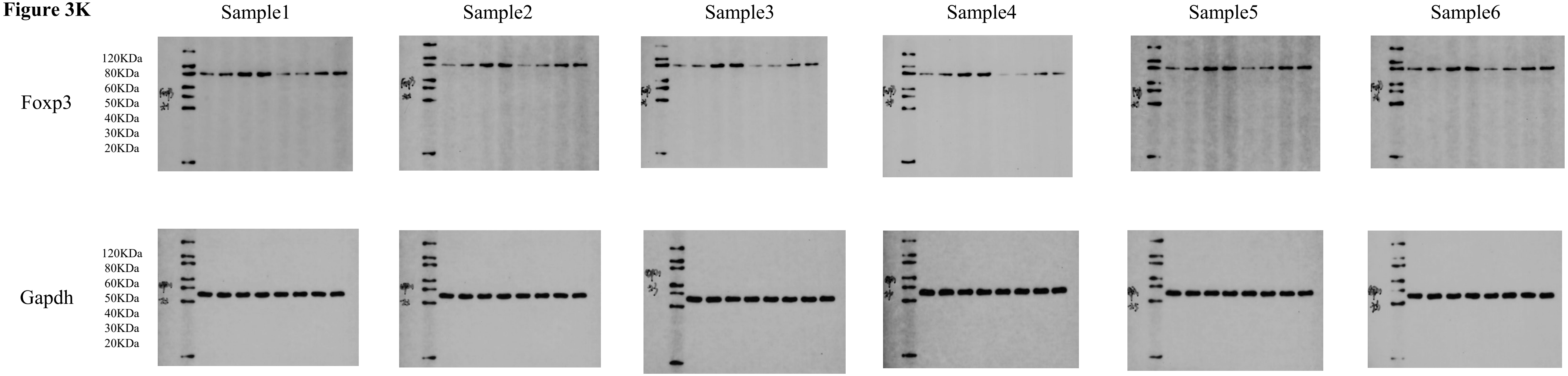
Sample1
Sample2
Sample3
Sample4
Sample5
Sample6
Foxp3
Gapdh
120KDa
80KDa
60KDa
50KDa
40KDa
30KDa
20KDa

Figure 4B

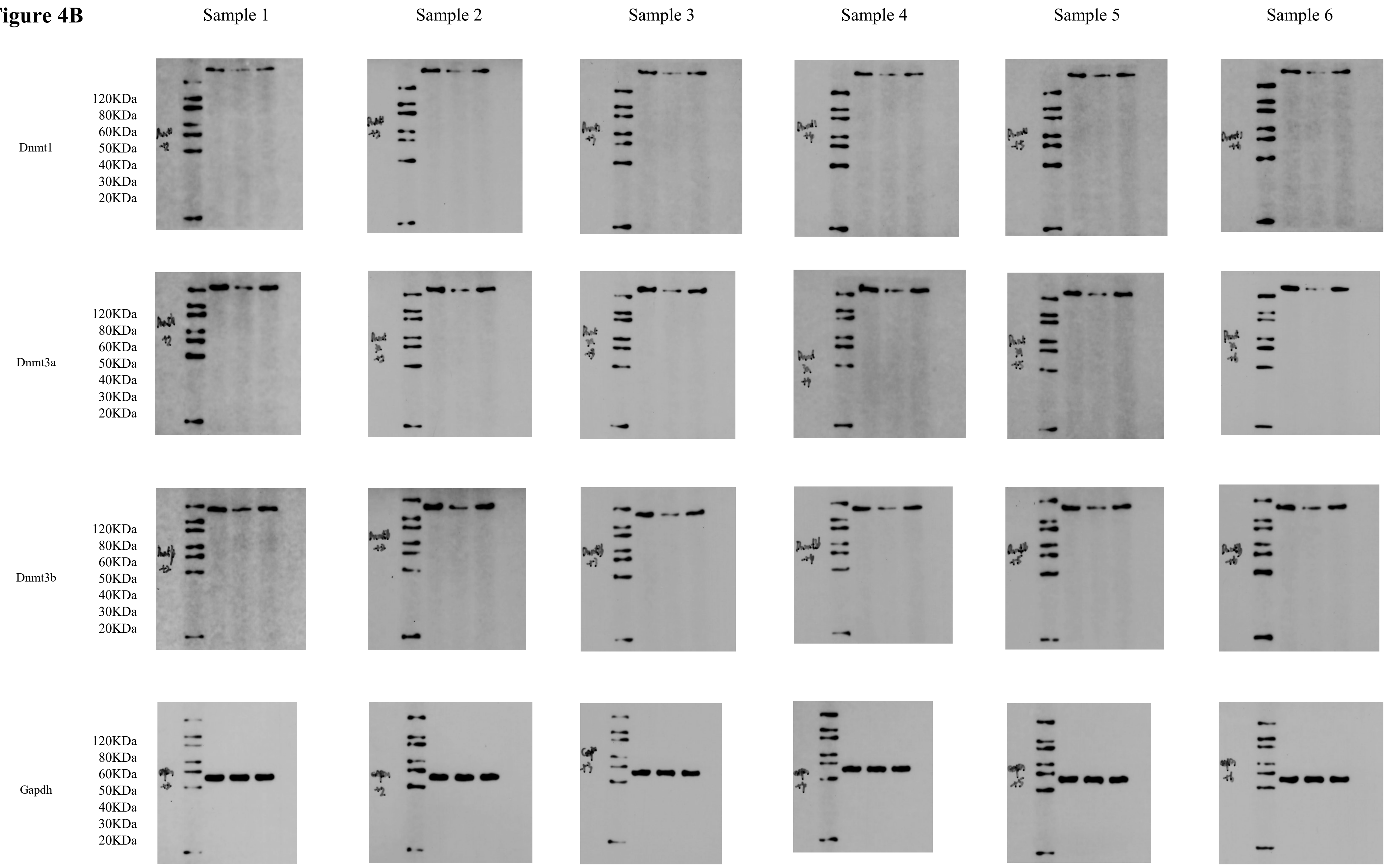
Sample 1
Sample 2
Sample 3
Sample 4
Sample 5
Sample 6
Dnmt1
Dnmt3a
Dnmt3b
Gapdh
120KDa
80KDa
60KDa
50KDa
40KDa
30KDa
20KDa

Figure 4D

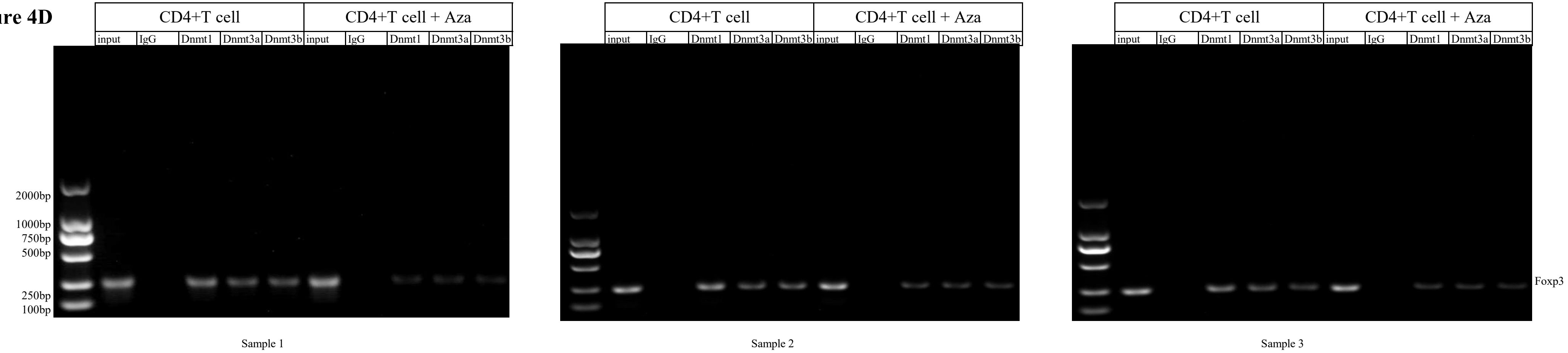
CD4+T cell
CD4+T cell + Aza
input
IgG
Dnmt1
Dnmt3a
Dnmt3b
2000bp
1000bp
750bp
500bp
250bp
100bp
Foxp3
Sample 1
Sample 2
Sample 3

Figure 4H

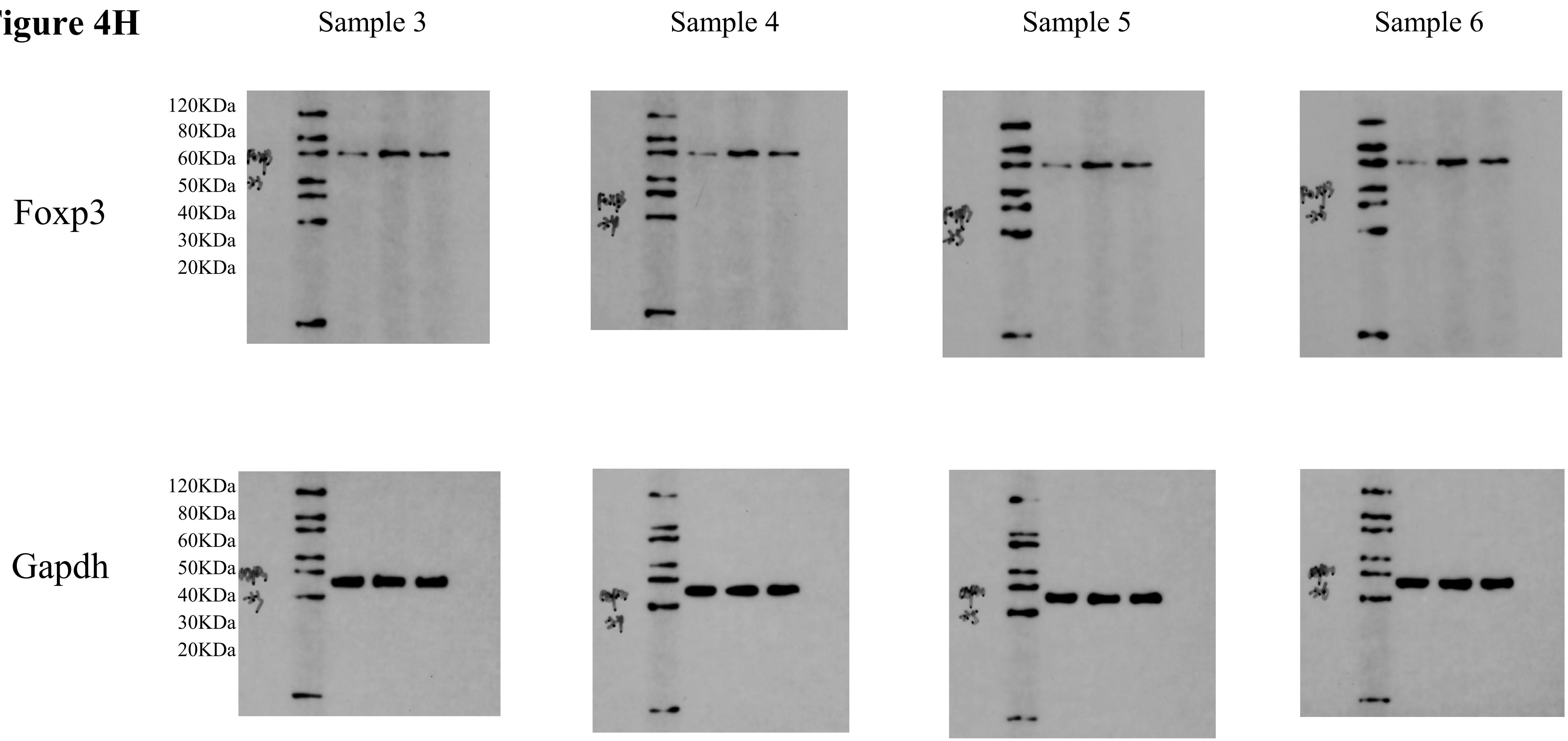
Sample 3
Sample 4
Sample 5
Sample 6
Foxp3
Gapdh
120KDa
80KDa
60KDa
50KDa
40KDa
30KDa
20KDa

Figure 5B

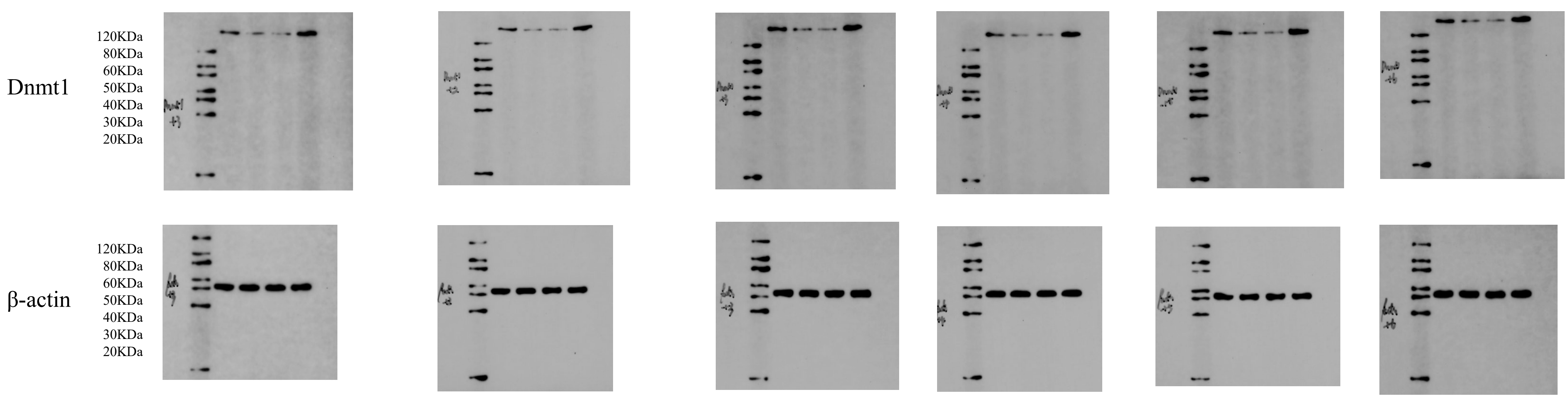
Sample 1
Sample 2
Sample 3
Sample 4
Sample 5
Sample 6
Dnmt1
120KDa
80KDa
60KDa
50KDa
40KDa
30KDa
20KDa
β-actin
120KDa
80KDa
60KDa
50KDa
40KDa
30KDa
20KDa

Figure 5G

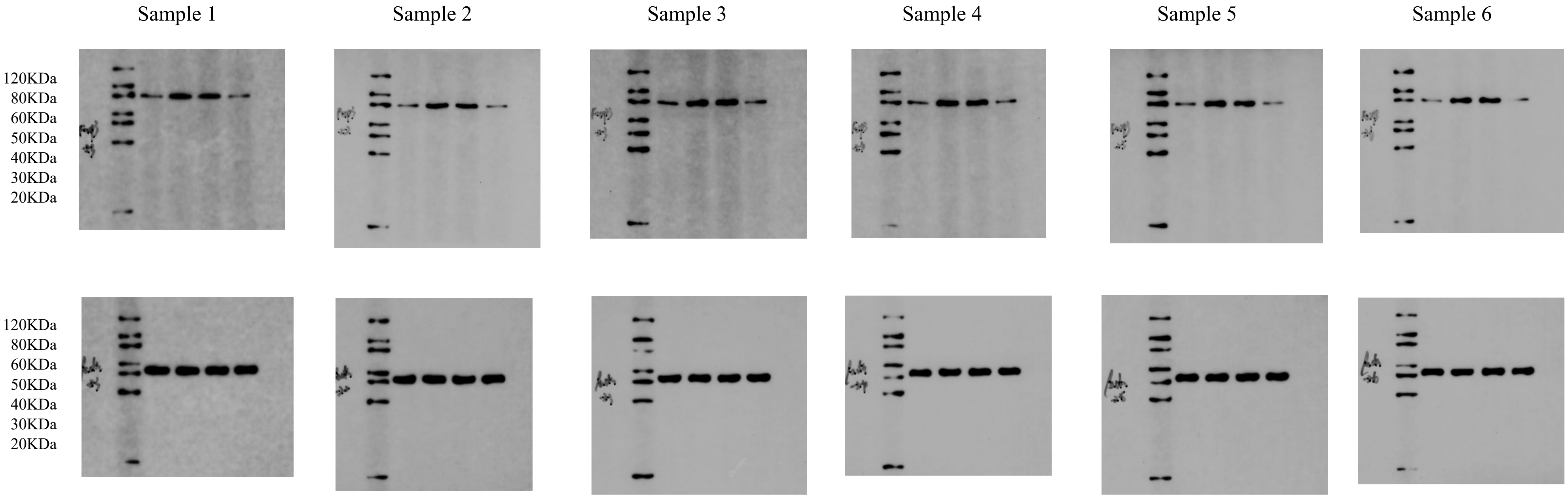

Sample 1
Sample 2
Sample 3
Sample 4
Sample 5
Sample 6
Foxp3
β-actin
120KDa
80KDa
60KDa
50KDa
40KDa
30KDa
20KDa
120KDa
80KDa
60KDa
50KDa
40KDa
30KDa
20KDa

Figure S2C

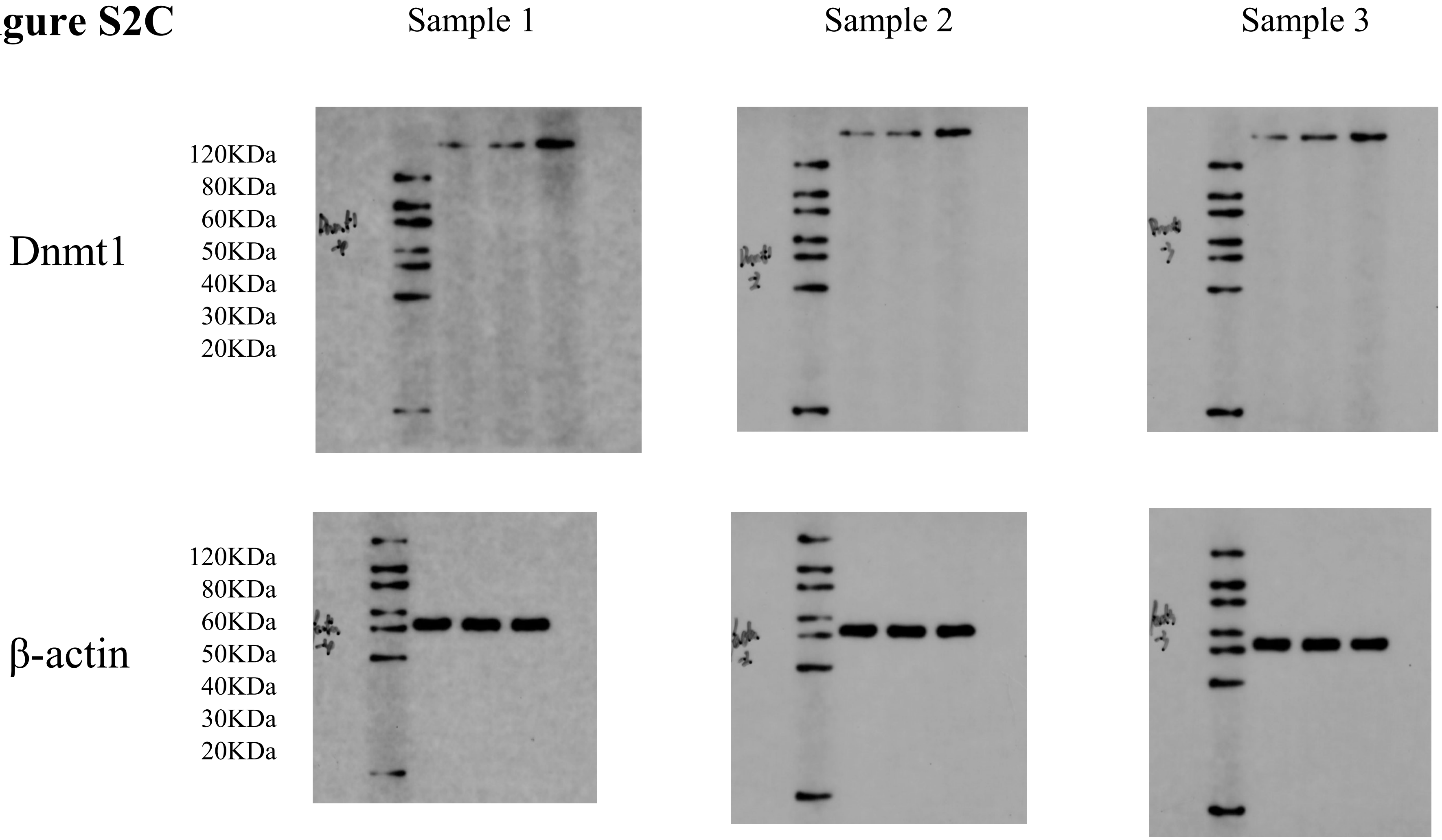
Sample 1
Sample 2
Sample 3
Dnmt1
β-actin
120KDa
80KDa
60KDa
50KDa
40KDa
30KDa
20KDa
120KDa
80KDa
60KDa
50KDa
40KDa
30KDa
20KDa

Figure S7

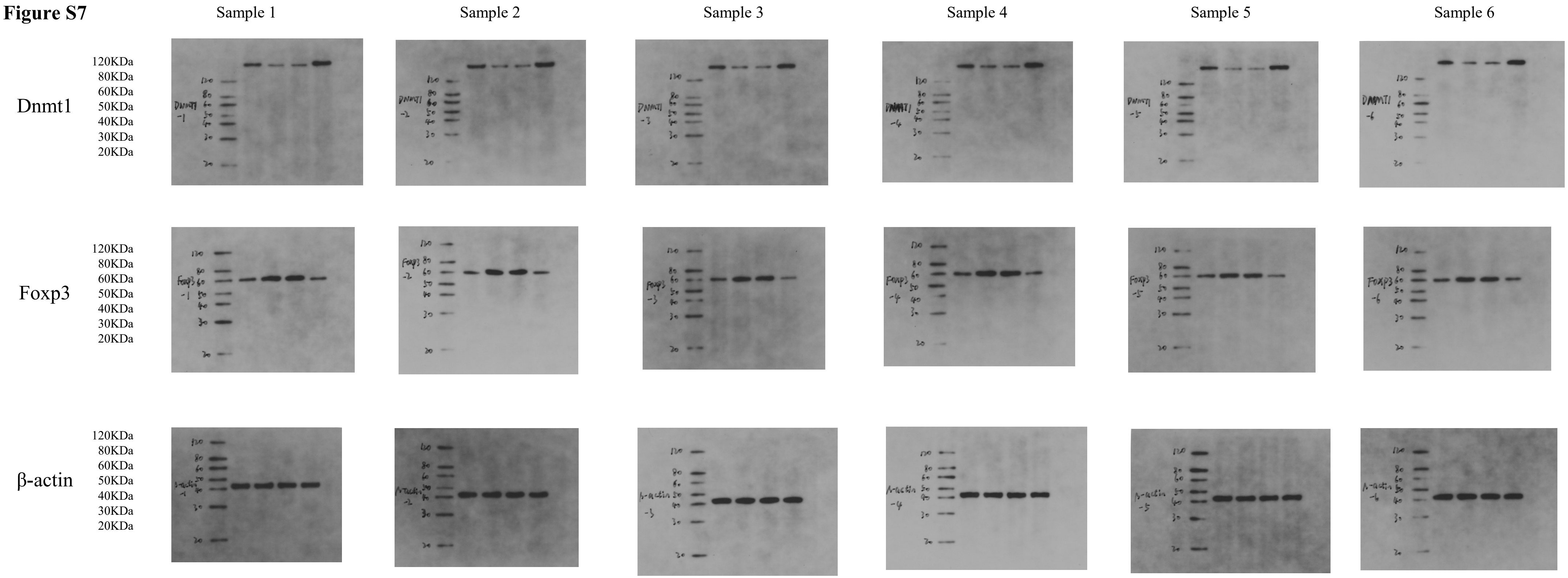
Sample 1
Sample 2
Sample 3
Sample 4
Sample 5
Sample 6
Dnmt1
Foxp3
β-actin
120KDa
80KDa
60KDa
50KDa
40KDa
30KDa
20KDa
